# The *Zea mays *mutants *opaque-2 *and *opaque-7 *disclose extensive changes in endosperm metabolism as revealed by protein, amino acid, and transcriptome-wide analyses

**DOI:** 10.1186/1471-2164-12-41

**Published:** 2011-01-18

**Authors:** Hans Hartings, Massimiliano Lauria, Nadia Lazzaroni, Raul Pirona, Mario Motto

**Affiliations:** 1 CRA - Unità di Ricerca per la Maiscoltura, Via Stezzano 24, 24126 Bergamo, Italy

## Abstract

**Background:**

The changes in storage reserve accumulation during maize (*Zea mays *L.) grain maturation are well established. However, the key molecular determinants controlling carbon flux to the grain and the partitioning of carbon to starch and protein are more elusive. The *Opaque-2 *(*O2*) gene, one of the best-characterized plant transcription factors, is a good example of the integration of carbohydrate, amino acid and storage protein metabolisms in maize endosperm development. Evidence also indicates that the *Opaque-7 *(*O7*) gene plays a role in affecting endosperm metabolism. The focus of this study was to assess the changes induced by the *o2 *and *o7 *mutations on maize endosperm metabolism by evaluating protein and amino acid composition and by transcriptome profiling, in order to investigate the functional interplay between these two genes in single and double mutants.

**Results:**

We show that the overall amino acid composition of the mutants analyzed appeared similar. Each mutant had a high Lys and reduced Glx and Leu content with respect to wild type. Gene expression profiling, based on a unigene set composed of 7,250 ESTs, allowed us to identify a series of mutant-related down (17.1%) and up-regulated (3.2%) transcripts. Several differentially expressed ESTs homologous to genes encoding enzymes involved in amino acid synthesis, carbon metabolism (TCA cycle and glycolysis), in storage protein and starch metabolism, in gene transcription and translation processes, in signal transduction, and in protein, fatty acid, and lipid synthesis were identified. Our analyses demonstrate that the mutants investigated are pleiotropic and play a critical role in several endosperm-related metabolic processes. Pleiotropic effects were less evident in the *o7 *mutant, but severe in the *o2 *and *o2o7 *backgrounds, with large changes in gene expression patterns, affecting a broad range of kernel-expressed genes.

**Conclusion:**

Although, by necessity, this paper is descriptive and more work is required to define gene functions and dissect the complex regulation of gene expression, the genes isolated and characterized to date give us an intriguing insight into the mechanisms underlying endosperm metabolism.

## Background

The developing maize (*Zea mays *L.) endosperm is a tissue primarily devoted to the accumulation of starch and proteins which, upon germination, provide nutrients for the germinating seedling. The investigation of regulatory constraints on endosperm development and on the synthesis of storage products provides an opportunity to understand the control of gene activity in eukaryotic cells [1].

Despite the apparent simplicity of the mature tissue, endosperm development is complex and combines several aspects regarding cell cycle regulation, cytokinesis, and cytoskeletal functions (reviewed by [2]). The first 7-12 days after pollination (DAP), characteristically involve cell division, after which the endosperm cells enlarge and as a result of several metabolic processes acquire storage proteins and starch [3]. Although the changes in storage reserve accumulation during maize grain maturation are well established, identifying key molecular determinants controlling carbon (C) flux to the grain and the partitioning of C to starch and protein remain elusive [1]. In fact, our understanding of how each pathway is controlled is complicated by the occurrence of multi-gene families encoding many of the enzymes in these biochemical pathways, the interconnectedness of these, and the strong influence of the environment on the amount and nature of the starch and protein synthesized [1].

Much of our current knowledge is based on biochemical assays of protein and enzymatic activities of starch and protein biosynthesis during caryopsis development. Zeins, the most abundant protein storage component in developing endosperms, are alcohol-soluble compounds with a characteristic amino acid composition, being rich in glutamine, proline, alanine, and leucine, and almost completely devoid of lysine and tryptophan [3]. Based on their solubility, genetic properties, and apparent molecular masses, zeins were classified into α- (19- and 22-kDa), β- (15-kDa), γ- (16-, 27-, and 50-kDa), and δ-zeins (10- and 18-kDa) that are encoded by distinct classes of structural genes [4]. The large α-zein component, accounting for > 70% of all zein proteins, is encoded by multiple active genes clustered in several chromosomal locations [5].

In this context, the analysis of maize mutants has facilitated the identification of many genes encoding starch synthetic enzymes and helped elucidate the process of starch formation [6]. Genetics has also played an important role by discovering a series of opaque endosperm mutants and demonstrating their effects on genes mediating zein deposition [1,7,8]. For example, the recessive mutations *opaque-2 *(*o2*) and *opaque-7 *(*o7*) induce a specific decrease in the accumulation of 22- and 19-kDa α-zeins, respectively.

The *o2 *mutation has been widely studied at the genetic, biochemical, and molecular level. *O2 *encodes a transcriptional regulator of the basic leucine zipper (bZIP) class that is specifically expressed in the endosperm activating the expression of 22-kDa α-zein and 15-kDa β-zein genes [9]. *O2 *also directly or indirectly regulates a number of other non-storage protein genes, including *b-32*, encoding a type I ribosome-inactivating protein, *cyPPDK1*, one of the two cytosolic isoforms of the pyruvate orthophosphate dikinase gene, and *b-70*, encoding a heat shock protein 70 analogue, possibly acting as a chaperonin during protein body formation [1]. *O2*, furthermore, regulates the levels of lysine-ketoglutarate reductase and aspartate kinase1 [10,11]. These broad effects suggest that *O2 *plays an important role in the developing grain as a coordinator of the expression of genes controlling storage protein, and nitrogen (N) and C metabolism [1].

Although the molecular basis of the *o7 *mutation is yet unknown, it was shown that this mutation, in addition to repressing the lower molecular weight α-zeins, drastically affects the development of maize endosperm due to a reduction in starch content. Moreover, the high content in *o7 *endosperms of non-protein N has suggested the existence in *o7 *of a block in the synthetic route leading to proteins similar to that observed for the starch modifying gene *shrunken4 *(reviewed in [12]).

To advance our understanding of the nature of the mutations associated with an opaque phenotype, we used nearly isogenic inbreds for *o2 *and *o7 *mutants, and for the double mutant combination *o2o7*, and compared their effects on protein synthesis and amino acid composition. In this study, to provide genome-scale information about gene expression patterns, we have also compared the profiles of gene expression in these mutants by cDNA microarray slides containing unique cDNAs expressed during kernel development. Microarray analysis provides an opportunity to examine the extent of changes in gene expression in mutants that are altered in metabolism. Classifying genes based on similarities or differences in transcript profiles within genotypes can confirm existing knowledge, lead to the dissection and revelation of novel mechanisms determining nutrient partitioning, and generate new unbiased hypotheses [13].

Recently, large databases of expressed maize genes have been made available (http://www.maizegdb.org  genoplante-info.infobiogen.fr; http://www.unicamp.br), and transcriptome analyses aimed at identifying genes involved in endosperm development and metabolism have been published [14,15]. Additionally, this technology was recently used to investigate a common mechanism that underlies several opaque-class kernel mutants [7]. The highly variable gene expression patterns they obtained made it difficult to identify common pathways that lead to soft endosperm texture. Our study extends their analysis by including the *o7 *mutation and the double *o2o7 *mutant, that appear useful in conjunction with the *o2 *mutation to i) identify and catalogue in endosperm the changes of genes involved in several metabolic pathways underlying the synthesis of storage reserves, ii) give new information about the effects of the *O7 *gene in endosperm metabolism in order to better understand its function in carbohydrate and protein syntheses, and iii) gain an insight into the complex gene system that integrates C and N metabolism in the developing endosperm.

## Results

### Effect of *o2*, *o7*, and *o2o7 *mutations on protein and amino acid compositions

**Figure 1:**
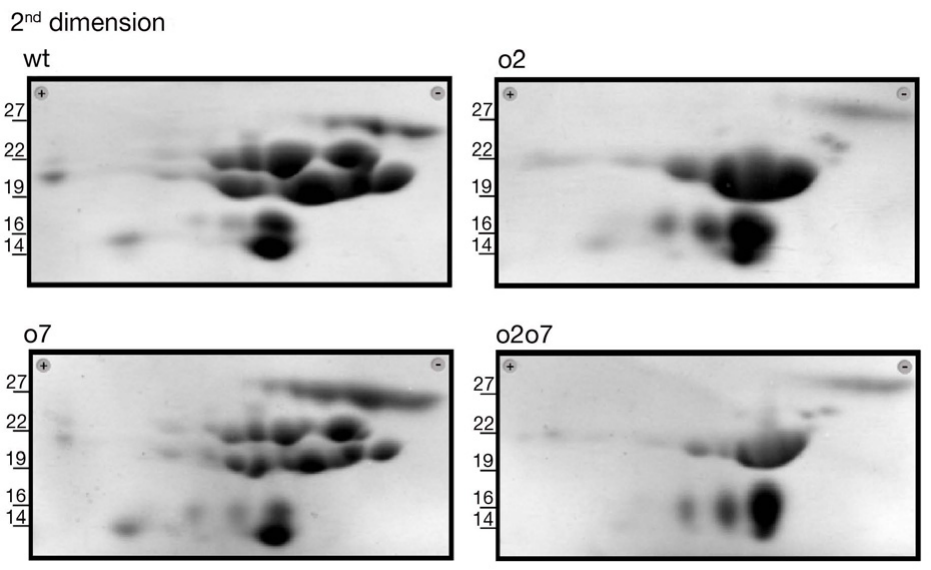
**Two-dimensional Analysis of Alpha-Zein Polypeptides**. Genotypes are indicated above each panel. Relative molecular weights derived from size standards are indicated as kDa values within each panel.

To verify whether the mutants analyzed exhibited qualitative and quantitative differences in protein composition compared to wild-type, we evaluated the protein and amino acid compositions of mature endosperm of the nearly isogenic A69Ywt, *o2*, *o7*, and *o2o7 *inbreds. Zein samples prepared from mature endosperm of the previous genotypes were compared in 2D-PAGE. In agreement with their apparent molecular masses on SDS-PAGE, 3 polypeptides were classified, based on their apparent molecular weight as 27-kDa γ-zeins, 5 as 22-kDa α-zeins, 6 as 19-kDa α-zeins, 3 as 15-kDa β-zeins, and 1 as a 16-kDa γ-zein (Figure 1). The two mutations decreased both the number and the accumulation of zein isoforms detected on 2D gels as compared to wild-type. The *o2 *mutation has a major effect on the 22-kDa class zeins as a complete reduction of most of these polypeptides was observed. In agreement with previous data, we confirm that the *o2 *mutation can also influence the accumulation of some members of the 19-kDa class [12]. Changes in the zein profile in A69Y*o7 *seeds were less evident. The *o7 *mutation decreased the amount of both 22-kDa and 19-kDa α-zeins as compared to wt. However, unlike previously reported, we did not find clear evidence of a specific polypeptide suppression mediated by the *o7 *mutation [16]. Finally, the zein pattern in the *o2o7 *background was strongly affected: the 22-kDa zein profile was nearly identical to the one observed in the *o2*, whereas polypeptides of the 19-kDa zein class were decreased both in amount and number. Taken together these data confirm an additive effect of the *o2 *and *o7 *mutations in reducing zein accumulation during endosperm development.

Table 1 provides data concerning the percentage contribution of the main N constituents present in the mature endosperm of the lines considered. With the exception of non-protein N, all N traits measured differed significantly, both in amount and composition, between wild-type and *opaque *mutants. The mutant alleles all reduced accumulation of total protein, although to varying extents, the effect being most marked in *o7 *(-28%). From these results it was possible to assess the importance of lysine-rich non-zeins with accuracy, because of the quantification of non-protein N and the exhaustive extractions of zeins. Thus, the ratio of non-zein content of the endosperm mutants compared with that of the wild-type varied from 1.1 to 2.2 for the single mutants, whereas for A69Y*o2o7*, a ratio of 2.0 was calculated. It was also evident that the effect of the *o2 *is more pronounced in reducing zein accumulation and increasing the other components than is *o7*. This behaviour is also evident in the *o2o7 *mutant, in which zein synthesis was most reduced, with a concomitant increase in albumin-globulins and glutelins, suggesting that in the double mutant both alleles are active in reducing zein synthesis additively.

**Table 1:** Protein and amino acid composition of mature A69Ywt, A69Y*o2*, A69Y*o7*, and A69Y*o2o7 *endosperm

Items	A69Ywt	A69Y*o2*	A69Y*o7*	A69Y*o2o7*
**Total protein**	**12.2**^a^	**10.8**^b^	**8.8**^c^	**9.7 **^b^

Albumins + globulins	**0.7**^a^	**2.0**^b^	**1.1**^c^	**2.2**^b^

Zeins *	**9.0**^a^	**3.8**^b^	**5.3**^c^	**3.2**^b^

Glutelins ^‡^	**2.5**^a^	**5.0**^b^	**2.5**^a^	**4.3**^b^

Non-protein nitrogen	0.7	0.7	0.6	0.6

Asparagine/Aspartate	**2.6 **^a^	**8.4**^b^	**6.2**^b^	**11.9**^c^

Threonine	1.6	2.4	2.6	3.0

Serine	2.3	2.8	2.8	3.2

Glutamate/Glutamine	**41.3**^a^	**34.0**^b^	**35.4**^b^	**24.6**^c^

Glycine	**1.3**^a^	**2.7**^b^	**2.4**^b^	**3.7**^c^

Alanine	6.4	5.0	5.1	5.1

Valine	**2.1 **^a^	**3.1**^b^	**3.2**^b^	**3.8 **^b^

Cysteine	1.0	1.8	1.5	1.9

Methionine	**0.6**^a^	**0.8**^b^	**1.1**^b^	**1.0**^b^

Isoleucine	**1.7**^a^	**2.4**^b^	**2.1**^b^	**2.5**^b^

Leucine	**16.2**^a^	**8.7**^b^	**9.3**^b^	**8.2**^b^

Tyrosine	1.0	1.4	1.2	1.5

Phenylalanine	**2.4**^a^	**3.0**^b^	**2.5**^b^	**3.0**^b^

Ornitine	0.0	0.1	0.0	0.1

Lysine	**0.9**^a^	**2.6**^b^	**2.2**^b^	**3.4**^c^

Histidine	**1.3**^a^	**1.9**^b^	2.3^b^	2.5^b^

Arginine	**1.4**^a^	**3.0**^b^	**2.5**^b^	**3.8**^c^

Proline	16.0	16.0	17.9	16.9

Protein and non-protein compositions are in percent of dry weight. Amino acid compositions are in percent of protein (w/w). * fractions Z1+Z2 of [49]; ^‡ ^fractions G1+G2 of [51].

^a,b,c ^Numbers followed by the same letters within a row are not significantly different at P ≤ 0.05 by standard analysis of variance.

The overall amino acid compositions of the single mutants *o2 *and *o7*, and of the double mutant combination *o2o7*, exhibited a rather similar pattern, although variation was observed of amino acid content in comparison to the wild type endosperms. Each of the single mutants had a high Lys content (>2.5-fold), whereas *o2o7 *had more that 3.5-times the amount present in the wild type. A similar shift, although less pronounced, was observed for Asx, and the other essential amino acids derived from the Asp pathway (*i.e*. Thr, Met and Ile) as well as for Gly, Val, His, and Arg. Among the amino acids reduced in the opaque endosperm mutants were Glx and Leu, the most abundant amino acids found in zein proteins. The reduction of these amino acids generally was inversely related to the increase in Lys, with the trend being more evident in the double *o2o7 *endosperm mutant.

### Microarray construction

Microarray slides were assembled using clones obtained from 20-part-normalized cDNA libraries representing the major events in endosperm development. 22,365 ESTs were sequenced, aligned, assembled into contigs using a similarity score of 90%, and annotated using BLASTX software. For each contig, the cDNA containing the largest transcript was identified. These, together with all singleton cDNAs (6719) were used to construct a unigene set of 8,950 sequences. The relative contribution of each cDNA library to the pool of identified ESTs is summarized in Table 2. It is notable that the distribution of ESTs across the original cDNA libraries was not uniform. The highest proportion of the sequences could be associated with endosperm tissue, the lowest with 8 days old embryo. EST sequences were analyzed with the BLAST2GO software (http://www.blast2go.org). In a first phase, homology searches using public domain non-redundant databases identified significantly homologous sequences for 48.4% of the ESTs considered. These ESTs represented 3,090 single hit and 1,240 multiple hit sequences.

**Table 2:** Relative contribution of each cDNA library to the pool of identified ESTs.

cDNA library	Contribution (%)
whole kernel (2 DAP)	5.3

embryo sac (3-4 DAP)	5.2

maternal tissue (3-4 DAP)	4.6

embryo (8 DAP)	4.3

whole kernel (6-8 DAP)	9.9

pedicelo-chalazal/basal endosperm transfer cells (12 DAP)	7.0

meristematic aleurone (12 DAP)	6.8

endosperm (10-28 DAP)	13.8

pericarp (10 & 21 DAP)	11.4

embryo (14 & 21 DAP)	10.4

aleurone (30 DAP)	2.0

germinated endosperm (20 DAP)	9.8

germinated aleurone (20 DAP)	9.7

The different cDNA libraries used and their relative contribution to the Zeastar unigene EST set are indicated.

**Figure 2:**
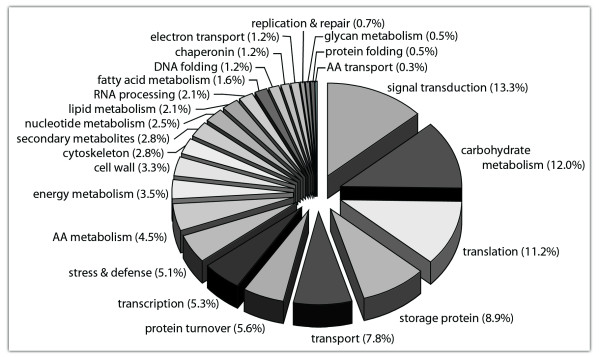
**Distribution of maize EST Unigenes amongst functional categories**. Gene-ontology categories were assigned to ESTs through curator-revised categorization. Eight thousand nine hundred and fifty endosperm preferred ESTs were classified. Gene-ontology terms (http://www.geneontology.org) were assigned based on similarity to known protein sequences in several databases using the BLAST2GO software (http://www.blast2go.org).The percentage of ESTs in each category is indicated next to the corresponding map sector.

In a second phase, an attempt was made to associate biological processes to each of the ESTs showing sequence homology using the gene ontology (G.O.; http://www.geneontology.org) and KEGG databases (http://www.genome.jp/kegg). Approximately 85% of these unigenes could be assigned a functional annotation, with the remainder (ca. 15%) having an ambiguous or unknown function. Figure 2 summarizes the assignment of the biological processes and molecular functions. Twenty-four distinct groups were identified to establish the complex regulatory hierarchies that exist to orchestrate the dynamic metabolic, transport, and control processes occurring in developing endosperm. This classification is consistent with the many functions of maize endosperm and is comparable with that reported by other workers [14,15]. It appears that our maize endosperm gene set is rather comprehensive and provides a good representation of the entire transcriptome including genes linked to accumulation of storage products and energy supply. More specifically, a large number of transcripts appeared to be involved in carbohydrate metabolism (12.0%), followed by those participating in storage protein synthesis (7.9%), translation (11.2%) and transcription (5.3%), nucleotide metabolism (2.5%), and RNA processing (2.1%). Among physiological processes, those transcripts implicated in protein turnover (5.6%), energy metabolism (3.1%), electron transport (1.2%), amino acid metabolism (4.4%), amino acid and sugar transport (7.8%), the latter being intrinsically linked to the accumulation of storage protein and starch, nucleic acid metabolism (2.5%), lipid (2.1%) and fatty acid metabolism (1.6%), and secondary metabolites (2.0%) were represented in our EST collection. Moreover, genes encoding for protein involved in cell wall (2.8%), cytoskeleton (2.8%), and stress and defence (5.1%) appear related to relevant cellular processes assigned in the functional classification. Finally, the assignment of other important classes of transcripts, such as DNA (1.2%) and protein folding (0.5%), transcription regulators (5.3%; mostly representing transcription factors), and signal transducers (13.3%) provides new perspectives for data mining and for studies of coordinated gene regulation in developing maize endosperm. Thus, ESTs corresponding to the majority of genes (or their alleles) are represented in the maize cDNA libraries constructed, and the use of the maize Zeastar unigene chip to examine endosperm gene expression appeared feasible.

### Effect of *o2*, *o7*, and *o2o7 *mutations on gene expression

The development of a Zeastar unigene chip made it possible to analyze the patterns of gene expression in the opaque mutants herein investigated. Microarray slides containing the entire Zeastar unigene set were hybridized with probes derived from endosperm tissue of normal, *o2*, *o7*, and *o2o7 *A69Y inbreds, harvested at 14 DAP, a developmental stage in which the synthesis of starch and storage protein is known to begin [1]. To reduce hybridization artefacts, all probes were labelled both with Cy3 and with Cy5 and used in dye-swapping experiments on series of three independent slides. The expression data obtained were assayed for consistency by performing ANOVA tests. Replicates appeared to be in general agreement; thus, we are confident that the alterations of the transcriptomes described here are consistent with the biology of endosperm development. Moreover, we selected a series of thirty clones, believed of particular interest and exhibiting distinct patterns of expression, for detailed analysis, using qRT-PCR to confirm the changes in expression levels determined using the arrays. RNAs isolated from the four genotypes were used as templates for amplification. The relative expression levels determined by qRT-PCR showed good agreement with those determined using microarrays (r = 0.91; see Materials). This degree of agreement is consistent to that observed for similar experiments. [e.g. 17]. Therefore, only the results of microarray analyses will be presented and discussed herein.

**Figure 3:**
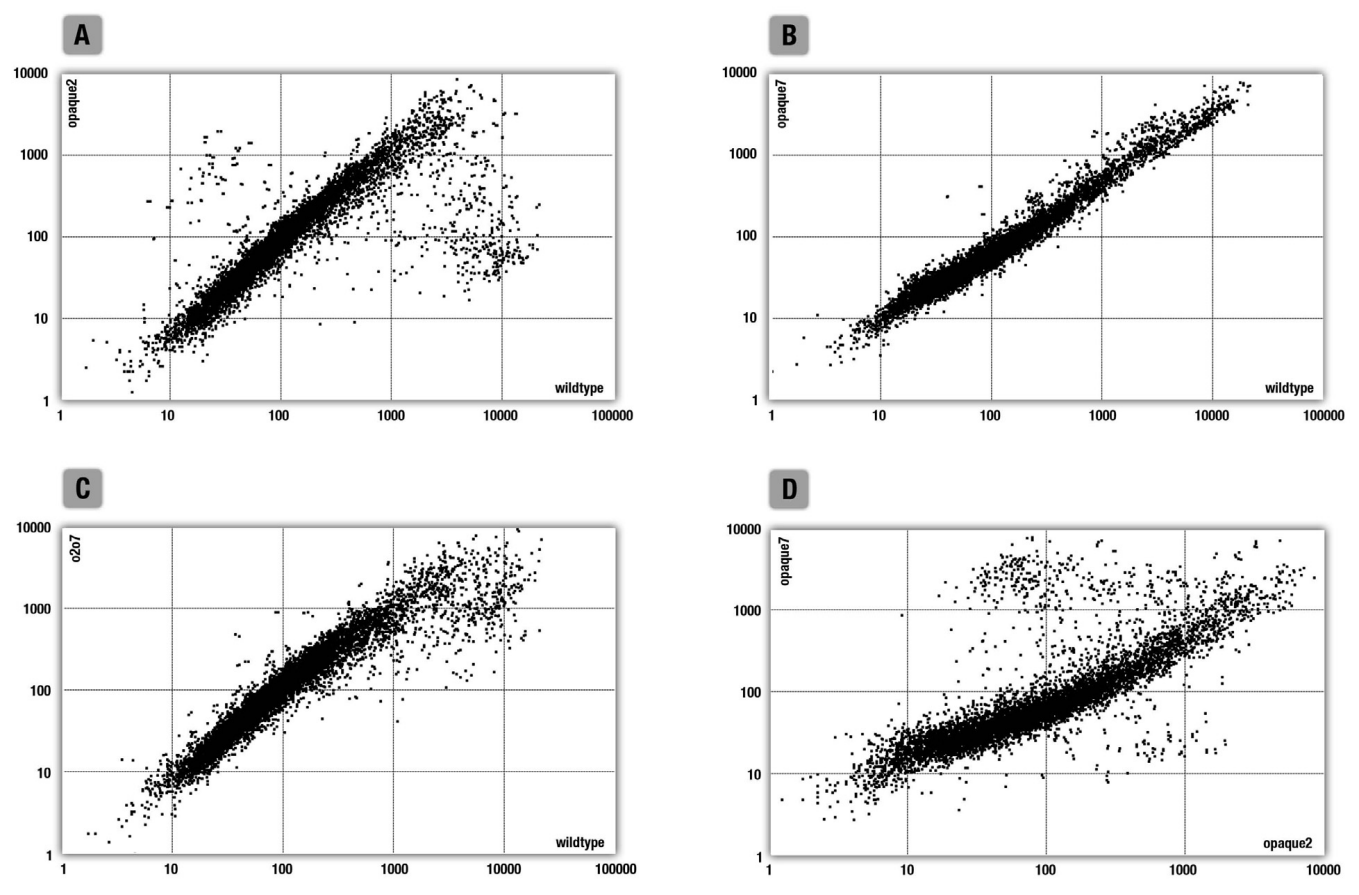
**Analysis of differential gene expression in A69Ywt, *o2*, *o7*, and *o2o7 *endosperms**. Signal correlation plots were used to examine mutant effects on the EST pool taken into consideration in endosperms at 14 DAP. (A) Signals derived for each EST in wild-type (x-axis) and *o2 *(y-axis) backgrounds were plotted using a logarithmic scale. Similar graphs were made to compare the expression of (B): wild-type (x-axis) vs. *o7 *(y-axis), (C): wild-type (x-axis) vs. *o2o7 *(y-axis) and (D): *o2 *(x-axis) vs. *o7 *(y-axis), respectively.

Average signal values derived from the four probes used were plotted using a logarithmic scale. Figure 3 shows plots of wt vs. *o2 *(3A), *o7 *(3B), and *o2o7 *(3C) signal values as well as *o2 *vs. *o7 *(3D) readings. Figure 3A clearly shows the prevalence of genes showing distinct expression patterns in the wt and *o2 *genotypes. Conversely, the wt and *o7 *genotypes show less evident differences in expression levels (Figure 3B). The *o2o7 *double mutant exhibits differences in expression patterns resembling those obtained for the *o2 *genotype, which, considering the low level of difference in expression level shown for the *o7 *genotype, is not unexpected. However, a plot of *o2 *vs. *o7 *expression levels, clearly shows the cumulative effect of both genotypes and reveals a large number of genes with distinct expression patterns (Figure 3D).

**Figure 4:**
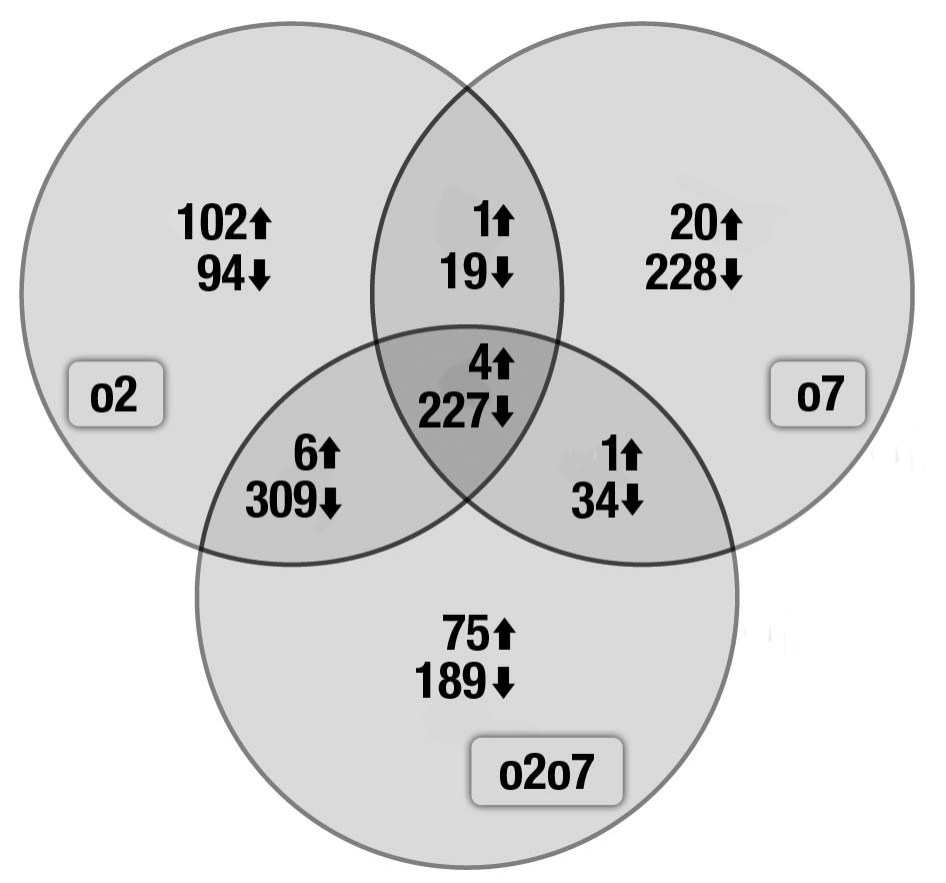
**Differential expression of ESTs in A69Y*o2*, *o7*, and *o2o7 *endosperms with respect to A69Ywt endosperm at 14 DAP**. Diagrams show the number of down- and up-regulated genes in the mutant endosperms with respect to wt endosperm.

Among the ESTs considered, 17.1% exhibited a down-regulated expression profile. The *o2 *mutation was associated with 649 down-regulated ESTs, 508 down-regulated ESTs were identified in the *o7 *background, whereas 759 ESTs showed a reduced expression pattern in the *o2o7 *background. Up-regulated expression profiles were found for 3.23% of the ESTs considered. One hundred and thirteen up-regulated ESTs were identified in the *o2 *background, 26 in the *o7 *background, and 86 in an *o2o7 *background. These results are summarized in Figure 4. Among the ESTs identified, 36.7% exhibited significant homology with sequences deposited in public databases and could be unequivocally associated with known biological processes. A complete list of differential gene expression detected in the mutant endosperms, for the various functional classes as described above, in comparison with wild-type, is available in Additional file 1, Table S1, while a selection of the most interesting up- and down-regulated genes is given in Table 3.

**Table 3:** Selected list of ESTs with significantly (P > 0.05) different mRNA levels.

EST ID	WT/o2 ratio	WT/o7 ratio	WT/o2o7 ratio	Description	Homology
**Amino acid metabolism**					

Zeastar-H39-C10	2.1			tryptophan synthase (**EC 4.2.1.20**)	EAZ38915.1

Zeastar-H10-F10	0.4	0.7	4.5	anthranilate phosphoribosyltransferase (**EC 2.4.2.18**)	EAZ03872.1

Zeastar-E16-C09	2.1	1.6	7.6	anthranilate synthase (**EC 4.1.3.27**)	EAZ30999.1

Zeastar-N02-C04	3.0			phosphoglycerate dehydrogenase (**EC 1.1.1.95**)	NP_001059330.1

Zeastar-C09-D04	2.4			cysteine synthase (**EC 2.5.1.47**)	NP_001105469.1

Zeastar-N17-E09	2.2	1.8	58.5	methionine synthase (**EC 2.1.1.14**)	ABK96186.1

Zeastar-N16-E03	2.7			s-adenosyl-l-methionine synthetase (**EC 2.5.1.6**)	CAJ45555.1

Zeastar-N10-D09	3.3	3.5	3.3	acetolactate synthase (**EC 2.2.1.6**)	Q41769

Zeastar-B03-C08	2.2	1.9	2.1	ketol-acid reductoisomerase (**EC 1.1.1.86**)	ABR25710.1

Zeastar-G05-L07	1.9			cytosine-specific methyltransferase (**EC 2.1.1.37**)	AAC16389.1

**Carbon metabolism and redox processes**					

Zeastar-N20-E07	1.7			citrate synthase (**EC 2.3.3.1**)	EAY81201.1

Zeastar-N12-A12	2.2			NADP-specific isocitrate dehydrogenase (**EC 1.1.1.42**)	NP_001043749.1

Zeastar-H33-C04	3.2			2-oxoglutarate dehydrogenase (**EC 1.2.4.2**)	CAH66433.1

Zeastar-E17-F12	2.2	3.3	1.8	succinate dehydrogenase (**EC 1.3.5.1**)	EAZ38613.1

Zeastar-B08-C05	2.3	2.9	2.7	malate dehydrogenase (**EC 1.1.1.37**)	AAK58078.1

Zeastar-E09-B12	2.0	2.5	2.8	lipoamide dehydrogenase (**EC 1.8.1.4**)	EAY96621.1

Zeastar-H28-H12	1.8			phosphoglycerate mutase (**EC 5.4.2.1**)	NP_001105584.1

Zeastar-L01-E03	2.1			pyruvate dehydrogenase (**EC 2.3.1.12**)	EAY90179.1

Zeastar-E04-F05		1.7		pyruvate kinase (**EC 2.7.1.40**)	NP_001065454.1

Zeastar-H27-B10		1.7		fructose-bisphosphate aldolase (**EC 4.1.2.13**)	EAZ10324.1

Zeastar-F02-C14	1.8	1.8	50.7	enolase (**EC 4.2.1.11**)	NP_001105896.1

**Starch metabolism**					

Zeastar-E02-E12	1.9			phosphoglucomutase (**EC 5.4.2.2**)	NP_001105703.1

Zeastar-C03-B05	2.5			granule binding starch synthase II (**EC 2.4.1.11**)	NP_001106039.1

Zeastar-H42-C03	1.9	2.4	3.2	sucrose phosphate synthase (**EC 2.4.1.14**)	NP_001105694.1

Zeastar-H13-H12	2.0			starch branching enzyme IIb (**EC 2.4.1.18**)	ABO25741.1

Zeastar-H26-F08			3.0	glucosyltransferase (**EC 2.4.1.13**)	EAZ44804.1

**Transcription and Translation**					

Zeastar-I06-G05	2.0			YABBY2-like transcription factor	EAY83940.1

Zeastar-B01-G12	0.5			MADS box protein	NP_001105332.1

Zeastar-C04-D08		2.0		MADS box protein	CAB85962.1

Zeastar-E06-D01	2.0			MADS box protein	NP_001105525.1

Zeastar-F04-L17		2.1	8.7	DNA binding protein opaque-2	NP_001105421.1

Zeastar-F01-L05			0.5	JAB1 protein	NP_001054112.1

**Signal transduction**					

Zeastar-F04-M10		1.9	9.1	auxin-binding protein	NP_001105353.1

Zeastar-H14-F07		2.0	2.1	auxin-binding protein	AAA33430.1

ZT-P21-4-F06	0.6			small GTP-binding protein RAB2	ABD59354.1

Zeastar-E02-H05	2.5			putative ser-thr protein kinase	NP_001042688.1

Zeastar-E08-A05	1.8			putative ser-thr protein kinase	AAP50960.1

Zeastar-E16-H11		0.4		D-type cyclin	EAZ04741.1

EST codes, WT over opaque expression ratios, EC descriptions, and databank homologies are indicated.

### Amino acid metabolism

**Figure 5:**
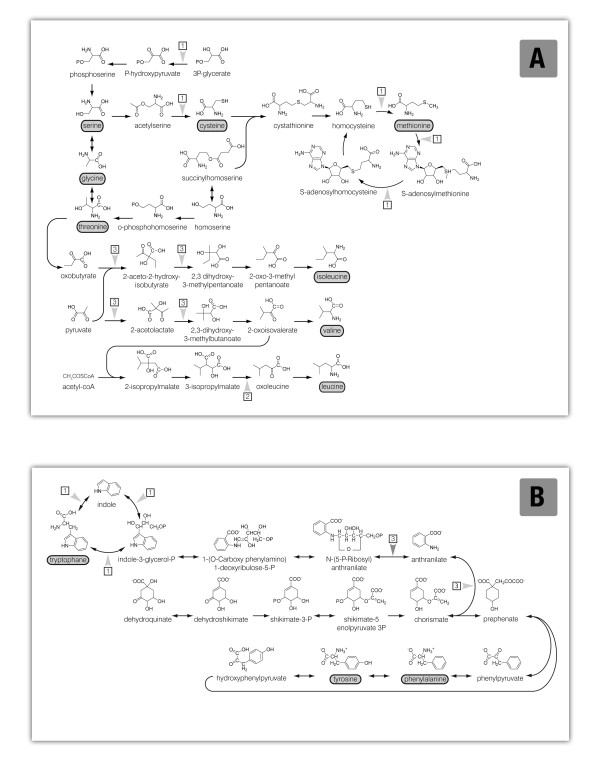
**Schematic representation of amino acid biosynthetic pathways**. Leu, Ile, Val, Thr, Met, Cys, Ser, and Gly (A) and Phe, Tyr, and Trp (B) biosynthesis pathways are indicated. Those steps associated with enzymes exhibiting an altered expression profile in the mutant endosperms analyzed with respect to the wt endosperm are marked. ESTs revealing an altered expression profile in A69Y*o2 *(1), A69Y*o7 *(2) endosperm, and both the A69Y*o2 *and A69Y*o7 *endosperms (3) are indicated.

Several ESTs homologous to enzymes involved in amino acid synthesis were differentially expressed in the *o2*, *o7*, and *o2o7 *endosperms. In particular, ESTs homologous to phosphoglycerate dehydrogenase (EC 1.1.195), cysteine synthase (EC 2.5.1.47), methionine synthase (EC 2.1.1.14), S-adenosylmethionine synthetase (EC 2.5.1.6), and a methyl transferase (EC 2.1.1.37), all enzymes involved in the Ser, Gly, Cys, and Met pathways were negatively affected in the *o2 *endosperm. However, neither of these showed a significantly altered expression level in the *o7 *and *o2o7 *endosperms (Figure 5A). Finally, the Ile, Val and Leu pathways were affected in all three lines. ESTs homologous to acetolactate synthase (EC 2.2.1.6) and ketolacid reductoisomerase (EC 1.1.1.86), and involved in the biosynthesis of these amino acids were significantly reduced in expression in all three backgrounds, while leucine dehydrogenase (EC 1.4.1.9) was significantly different from wt only in the o7 endosperm (Figure 5A).

ESTs homologous to enzymes involved in tryptophan synthesis were affected in *o2 *endosperm. Tryptophan synthase (EC 4.2.1.20) homologues showed a significant reduction of expression in *o2 *endosperms, while anthranilate phosphoribosyl transferase (EC 2.4.2.18) and anthranilate synthase (EC 4.1.3.27) homologous ESTs were found to be differentially expressed in all three mutant backgrounds. The former showed a significant reduction of its expression level, while the latter appeared up-regulated by 50% (Figure 5B).

### Carbon metabolism and redox processes

Maize is an autotrophic organism that only needs minerals, light, water and air to synthesize organic compounds to grow, however, endosperm is a heterotrophic organ. A large proportion of its proteins support primary metabolic processes and synthesis of more or less complex molecules such as nucleotides, amino acids, carbohydrates, lipids and secondary compounds. Accordingly, alterations in the expression levels of several genes encoding enzymes involved in these processes are expected in this study.

**Figure 6:**
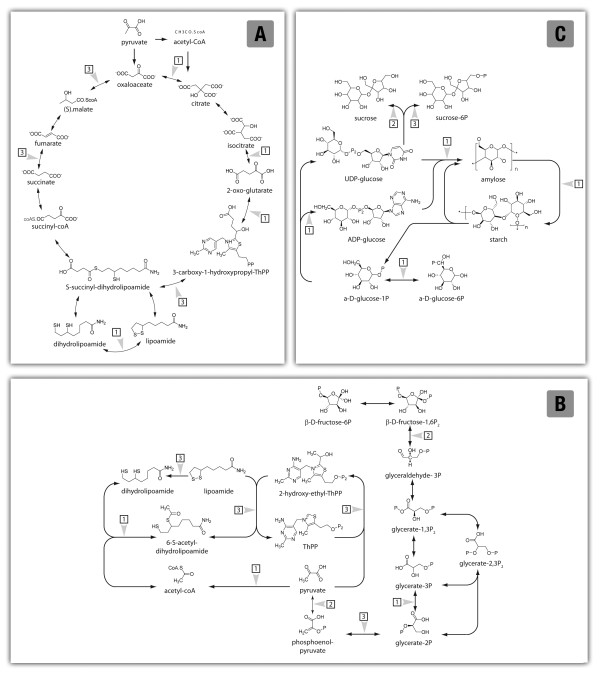
**Schematic representation of metabolic pathways**. The citrate cycle (A), glycolysis (B) and starch (C) metabolisms are represented. Steps associated with enzymes exhibiting an altered expression profile in the mutant endosperms analyzed with respect to the wt endosperm are marked. ESTs revealing an altered expression profile in A69Y*o2 *(1), A69Y*o7 *(2) endosperm, and both the A69Y*o2 *and A69Y*o7 *endosperms (3) are indicated.

A large set of ESTs exhibiting differential expression amongst the lines analyzed showed sequence homology with enzymes involved in C metabolism, including the trichloroacetic (TCA) cycle and glycolysis. In particular, seven ESTs homologous to TCA cycle related enzymes were identified all of which were down-regulated. Four of the ESTs were down-regulated only in the *o2 *endosperm. These are related with oxaloacetate to citrate (citrate synthase - EC 2.3.3.1), isocitrate to 2-oxo-glutarate (isocitrate dehydrogenase - EC 1.1.1.42) and 2-oxo-glutarate to 3-carboxy-1-hydroxypropyl-ThPP and S-succinyl-dihydrolipoamide (oxo-glutarate dehydrogenase - EC 1.2.4.2) inter-conversions. The remaining ESTs, which could be associated with succinate to fumarate (succinate dehydrogenase - EC 1.3.5.1), malate to oxaloacetate (malate dehydrogenase - EC 1.1.1.37), and lipoamide to dihydrolipoamide (dihydrolipoamide dehydrogenase - EC 1.8.1.4) interconversions, were differentially expressed in all three backgrounds (Figure 6A). Furthermore, ESTs associated with nine steps of glycolysis and exhibiting significant lowered expression patterns were identified. Phosphoglycerate mutase (EC 5.4.2.1), pyruvate ferredoxin oxidoreductase (EC 1.2.1.51), and dehydrolipoamide acetyltransferase (EC 2.3.1.12) were found in the *o2 *background only; pyruvate kinase (EC 2.7.1.40) and fructose biphosphate aldolase (EC 4.1.2.13) were found in the *o7 *endosperm, while ESTs homologous to dehydrolipoamide dehydrogenase (EC 1.8.1.4), pyruvate dehydrogenase (EC 1.2.4.1), and enolase (2-phospho-D-glycerate hydratase; EC 4.2.1.11) exhibited a reduced expression in all three backgrounds (Figure 6B). Furthermore, several genes involved in the redox status such as cytochrome C oxidase/reduction, thieredoxin and pyrophosphatase were strongly negatively affected in the opaque mutations, while a H^+^-transporting ATPase and a thiosulfate sulfurtransferase were greatly increased in *o2 *and *o7 *endosperms, respectively.

### Starch metabolism

Our profiling assays identified six differentially expressed ESTs exhibiting sequence homology with starch and sucrose metabolism related enzymes. ESTs homologous to enzymes catalyzing the inter-conversion from α-D-glucose-6P into α-D-glucose-1P (phosphogluco mutase - EC 5.4.2.2), from α-D-glucose-1P into ADP-glucose (glucose-1P thymidylyl transferase - EC 2.7.7.24), from ADP-glucose into starch (starch synthase - EC 2.4.1.11) and from amylose into amylopectin (amylase isomerase - EC 2.4.1.18) were down-regulated in expression in the *o2 *background only. UDP-glucose to sucrose conversion (sucrose synthase - EC 2.4.1.13) appeared down-regulated in the *o7 *background, while UDP-glucose to sucrose-6P conversion (sucrose phosphate synthase - EC 2.4.1.14) appeared down-regulated in all three backgrounds (Figure 6C).

### Storage protein synthesis

As expected, storage protein synthesis was greatly affected in the mutant backgrounds analyzed. In the *o2 *background, a reduction of the 22-kDa α-zein transcription pool was observed, while a concomitant increase of 10-, and 50-kDa γ-zein transcripts was seen. The *o7 *endosperm showed a marked reduction of 19-kDa α-zein transcription levels, as well as a reduction of 10-, 27-, and 50-kDa γ-zeins. The transcription level of the 18-kDa zein class appeared increased in this background. Finally, the *o2o7 *endosperm showed a reduced transcription level of the 10-kDa γ-, 19- and 22-kDa α-, and 27- and 50-kDa γ zein gene pools.

### Transcription and translation

A series of ESTs homologous to genes involved in gene transcription and translation processes showed variation in the expression patterns analyzed. In particular, three putative MADS-box domain transcription factors (TFs) were identified in the *o2 *background as well as two G-box binding factors and a YABBY2 factor, a member of the YABBY family of TFs, were down-regulated in *o2*. The *o7 *endosperm showed differential expression of a putative MADS-box gene, a putative MYB family transcription factor and a homologue of the OCL5 DNA-binding homeobox protein. It was interesting to note that in the *o7 *endosperm mutant the expression of the transcriptional regulator *O2 *is significantly down-regulated. Additionally, ESTs homologous to the JAB1 protein (a putative JUN activation domain binding protein) and to a putative G-box binding factor showed altered expression in the *o2o7 *background. All ESTs mentioned showed down-regulation. It was also evident from our data that ESTs encoding proteins such as histone H2A, H2B and H3, H4, which are involved in chromatin function, were down-regulated.

### Signal transduction

In the endosperm mutants, particularly in *o2 *and *o2o7*, the amounts of several transcripts involved in signalling by phosphorylation/dephosphorylation were reduced. The repressed genes encoded putative receptor kinases, protein-kinase-like proteins, Ser/Thr protein phosphatases, and auxin-binding proteins. It is known that these proteins play pivotal roles in regulating and coordinating aspects of metabolism, cell growth, cell differentiation, and cell division (review in [18]). The switching on and off of these genes is crucial for their correct function. Our results also indicate that in the o2 endosperms the level of transcript encoding a protein phosphatase and a small GTP binding protein RAB2 were increased. Similarly, in the *o7 *and in *o2o7 *endosperms we noted an increase in a D-type-cyclin and in a putative nitrogen-activated protein kinase, respectively.

### Protein synthesis, turnover, and destination

The protein synthesis machinery plays an important role in endosperm development and its biosynthesis entails the co-expression of a number of specific proteins. In the protein synthesis categories, mainly the ESTs encoding putative ribosomal proteins, translation initiation and elongation factors showed, to various extents, a reduced transcription level in the mutant endosperms compared to wild-type endosperm. For example, ESTs homologous to translation initiation factors 1β, 3α, 4α, and 5α and to elongation factor 1β were reduced in expression in all endosperm mutants considered.

Potentially also very interesting is the fact that several genes involved in protein degradation (ubiquitin pathway, a range of proteases, and heat shock protein genes) appeared repressed in the mutant endosperms, with the exception of some ESTs (pre-pro-cysteine proteinase, 26S proteosome regulatory subunit, particle-triple-A ATPase subunit 3, and serine peptidase) that are activated in the *o2 *and *o2o7 *endosperms. Protein degradation can be part of the normal protein turnover process, but can also play an important role in the control of endosperm development or can be part of an ubiquitin ligase complex involved in signalling via protein degradation.

### Fatty acids, lipid, cell wall and cytoskeleton synthesis

The expression of some genes annotated as involved in fatty acid biosynthesis and oil storage were repressed in all the endosperm mutants. Among the secondary compound category involved in cell wall lignification or cell wall polysaccharide synthesis, a range of genes encoding enzymes involved in cell wall growth (e.g. encoding an endo-1,4-β-glucanase *Cell1*; a cellulose synthase; a hydroxyproline rich glycoprotein) involved in the synthesis of cellulose are poorly expressed in the endosperm of the mutants. Considering the cytoskeleton, in both endosperm mutants the down regulation of genes involved in tubulin and actin biosynthesis were observed.

### Transport and stress

The transcript levels of several genes involved in amino acid, lipid, protein and membrane transport were down-regulated in the opaque mutants. Furthermore, down-regulation of the various transcripts encoding temperature-stress, inducible proteins, and pathogenesis related proteins were noted in the mutant endosperms. In *o2 *endosperm a putative low-temperature and salt responsive protein and putative Pi starvation-induced proteins were significantly induced, while a heat shock protein HSP101 and a wound-induced protease inhibitor were increased.

## Discussion

As highlighted before, endosperm growth and development is a complex phenomenon that may be driven by the coordinate expression of numerous genes. Approaches using spontaneous and induced mutants allow the characterization of the complex underlying gene expression system integrating carbohydrate, amino acid, and storage protein metabolisms, and operating during endosperm growth and development. The current work confirms other studies carried out on the *o2 *and *o7 *mutations (reviewed in [1]), in revealing considerable qualitative and quantitative differences between the endosperm protein assets of these genotypes. The mutant alleles at these loci are both recessive, and when homozygous, repress mainly the higher and lower molecular weight α-zein subunits, respectively, with an accumulation of albumins, globulins, and glutelins. The major shift in expression from zein to non-zein genes is consistent with changes in the patterns of protein synthesis in the endosperm. Moreover, in the *o2o7 *double mutant, the alleles act additively and possibly independently on zein synthesis. It is very likely that the different genetic backgrounds used in the various experiments may have an impact on storage protein synthesis by considering the exceptional haplotype variability in maize genomic regions containing zein genes (see e.g. [5,13]). Our data confirm previous findings that the *o2 *and *o7 *mutations nearly double the Lys content in maize endosperm and, thereby, significantly improve the nutritive quality of the grain ([19], and references therein). Furthermore, we found evidence in the opaque mutants herein investigated for high levels of other essential amino acids derived from the Asp pathway (i.e. Thr, Met and Ile-Leu), as well as for Arg and Gly.

To better clarify the role that *O2 *and *O7 *play in endosperm gene expression and to investigate their possible interactions, we have mRNA profiled wild-type, *o2*, *o7*, and *o2o7 *mutant endosperms. The ability to concurrently profile the expression of many genes in a tissue provides a powerful tool for comparing endosperm mutants with their wild-type counterparts to understand their functional role in metabolic processes. Although changes in gene expression (mRNA level) do not necessarily lead to changes in protein levels or to changes in developmental processes, the importance of transcription as a control point in development is well established for both plant and animal systems [20].

In this study, the profiling of endosperm transcripts was obtained with the Zeastar unigene set, based on the sequence information of >7,200 maize genes, mainly derived from maize endosperm and covering a wide range of metabolic pathways and cellular and physiological processes. The number of genes identified in our study was consistent with other reports suggesting that at least 5,000 and 4,500 to 8,000 different genes could be expressed, respectively in maize and wheat endosperms cDNA libraries [14,21]. These numbers were also considered a minimal estimate in a similar investigation previously reported in maize [15]. To validate the observed alterations in developing endosperms, we have used qRT-PCR, which confirmed that the observations regarding transcript accumulation were accurate and consistent with the findings of other laboratories undertaking similar studies (for an overview see [22]). They also take into account sources of variation inherent to microarray experiments [23]. Thus, we are confident that the alterations of the transcriptomes described here are consistent with the biology of endosperm development and are both real and significant.

In agreement with previous results regarding the analysis of a range of opaque mutants (including *o2 *but not *o7) *with an Affimetrix GeneChip, our transcriptomic analyses demonstrate that the *o2 *and *o7 *mutants here investigated are very pleiotropic and influence several metabolic processes occurring in the developing endosperm [7]. The degree of the pleiotropic effect varied among the mutants: *o7 *has the smallest effect on global patterns of gene expression, consistent with the relatively small differences in protein and amino acid composition in this mutant compared to the wild type. By contrast, the large changes in protein and amino acid synthesis in *o2*, replicated also in the *o2o7 *double mutant, are associated with large changes in the patterns of gene expression.

Although, the type of microarray analysis discussed in this paper does not distinguish between direct and indirect effects, making it difficult to conclude whether and how a TF interacts with a potential target gene, the analyses of the changes in the transcription profiles of the *o2 *and *o7 *mutants allow us to formulate predictions regarding the biological role of these loci in endosperm metabolism. First, our findings are consistent with the role of *O2 *as a transcriptional activator. In fact, the O2 protein is known to regulate the expression of genes that encode the 22-kDa α-zein gene family [24]. Moreover, it controls the expression of other non-storage protein genes (e.g. [7,8] and additional files online). Second, one of the pathways affected by O2 activity is amino acid biosynthesis. It has been shown that O2 regulates the levels of lysine-ketoglutamate reductase, aspartate kinase, acetohydroxyacid synthase, an enzyme catalyzing the first common step in the synthesis of branched chain amino acids (BCAA), and cyPPDK1, a key regulator of the glycolytic pathway, linked to C and amino acid metabolism and to the starch-protein balance [10,11,25-27]. This associated with its structural and functional similarity to GCN4, a general transcription factor regulating amino acid biosynthesis in yeast (*Saccharomices cerevisiae*;), reinforces the hypothesis that *O2 *may be indeed involved in general amino acid control in maize endosperm [28,29]. In the current study, the transcription levels of various genes encoding key enzymes involved in amino acids were significantly affected in the *o2 *mutant. *O7 *also influences the expression of some genes of the amino acids biosynthesis, but only in few cases the mRNAs affected are the same that are up- or down-regulated in the *o2 *mutant, suggesting that the *O2 *and *O7 *factors act on specific target genes. Among the pathways affected by *o2 *and *o7 *mutants are those leading to the synthesis of the aromatic (Phe, Trp, and Tyr), Asp-derived, and BCAA aminoacids. These pathways are deeply interconnected both in terms of C precursor supply and of allosteric interactions [30]. A complex interplay of regulators controls the metabolic flow through the aromatic, Asp and BCAA-pathways, which includes feedback inhibitors of regulatory enzymes [31,32]. Moreover, alterations in enzymes affecting amino acid metabolism have been shown to have pleiotropic effects on free amino acid levels in plant tissues. For example, Frankard *et al*. found that a mutation in a key enzyme in the Asp-pathway, a feedback-insensitive aspartate kinase mutant in tobacco, not only has a higher level of amino acids derived from the Asp pathway, but other pathways as well [33]. Guillet *et al*. reported that the alteration of Trp and Tyr levels in transgenic tobacco leaves affects the level of Trp, as well as the aliphatic amino acids Met, Val, and Leu [34]. Furthermore, there is evidence indicating that glutamate is an allosteric regulator of phosphoenolpyruvate carboxilase (PEPC) and pyruvate kinase (PK) generating, respectively, oxalacetate and pyruvate, that, in addition to PEP, are intermediate metabolites that play a central role in plant primary and secondary metabolisms, including amino acids biosynthesis [35].

Our results further indicate that *o2 *and *o7 *alter gene expression in a number of enzymatic steps in the TCA cycle and glycolysis pathway that are of central importance for the amino acid metabolism in developing seeds. Therefore, both *O2 *and *O7 *are expected to induce multiple effects on endosperm metabolism by modulating the glycolytic and TCA pathways. An alteration in the expression patterns of glycolytic and TCA enzymes in developing endosperm is related to the multiple pathways and demands on central enzymes of intermediary metabolism. In addition, during endosperm development, the active use of C precursors and energy from glycolysis is required for rapid cell division, and in the accumulation phase these resources may simply be redirected to storage compound syntheses. Regarding glycolysis, evidence indicates that both regulatory and structural genes influence the glycolytic pathway [36]. Because regulators of glycolysis have not been mapped in maize, it is also of interest to compare the activity of several key enzymes in this pathway. However, a systematic characterization of such enzymes will be necessary before any inferences are warranted.

In this context a further interesting observation resulting from this study regards the altered expression of several enzyme encoding genes, e.g. PK, pyruvate dehydrogenase, and enolase, involved in pyruvate metabolism, suggesting that *O2 *and *O7 *are, likely indirectly, implied in the regulation of this metabolite. Recent results, obtained by constitutive over-expression of the maize TF *Dof*, a member of the Dof (DNA-binding with one finger) TFs unique to plants, in transgenic *Arabidopsis *was directly associated with the PEPC gene expression, leading to a marked increase in acid contents, and a reduction of glucose [37]. In addition, transgenic expression in potato of a PEPC insensitive to feedback inhibition by malate resulted in a shift of C flux from soluble carbohydrates and starch to organic acids and amino acids [38], implying the ultimate link between C and N metabolism. Thus, the *o2 *and *o7 *mutation may lead to an increased level of pyruvate by down-regulating genes encoding enzymes involved in the pyruvate metabolism providing a link between C and N partitioning.

A further outcome from our work concerns the down regulation observed in the *o2o7 *double endosperm mutant, in comparison to wild-type, of genes encoding auxin-binding proteins. The phytohormone auxin regulates a wide variety of plant developmental programs through various regulatory mechanisms, including auxin-binding proteins [39]. For example, in maize the synthesis of a number of seed storage proteins has been shown to be subjected to regulation by phytohormones [40]. Moreover, recent evidence indicates that a reduced accumulation of auxins in the maize defective endosperm*-B18 mutant, due to down regulation of *Pinformed1*, a member of the PINFORM family of auxin efflux carriers, leads to a reduction in dry matter accumulation in the seed [41]. Similarly, the cell wall invertase-deficient *miniature1 (mn1) *mutant exhibits several pleitropic changes, including a reduction in kernel mass and a detrimental effect on auxin levels throughout kernel development, indicating that developing seeds may modulate growth by altering tryptophan-dependent auxin biosynthesis in response to sugar concentration [42]. This has suggested a potential cross talk between sugar and auxin pathways. It is tempting to speculate, on the basis of the present and previous studies on the *o2 *and *o7 *mutants, indicating a reduction in kernel mass and an altered sugar metabolism, that a drastic imbalance of the sugar metabolism in the *o2o7 *endosperm mutant may be the cause of the observed down regulation of enolase and auxin-bindin protein gene expression [1]. However, further research on these versatile signaling switches will be needed to clarify this point.

A close examination of the expression patterns of genes involved in sugar and starch metabolism shows that both the *o2 *and *o7 *mutations create perturbations in the hexose/sucrose metabolism. It has been reported that sugars, such as glucose and sucrose, can act as signals to trigger changes in the expression of a broad range of genes, including genes associated with C and N metabolism, signal transduction, and post-transcriptional modification of proteins [43-45]. In addition, Price *et al*. found that a large number of stress-responsive genes were also induced by glucose, indicating a role of this sugar in the environmental response [44]. Moreover, one group of genes consistently affected in the opaque mutants, has been implicated routinely in stress responses. Hunter *et al*. (and references therein), reported that the opaque mutations disrupt the organization of α- and γ-zeins in the protein body and lead to the increased expression of cellular stress response genes, such as those encoding molecular chaperones, cell wall proteins, and wound- and pathogen-activated proteins[7]. In this respect, Segal *et al*. found that RNAi-mediated silencing of the genes encoding the 22-kDa α-zeins caused the mature endosperm to become starchy, indicating that the reduced synthesis of 22-kDa α-zeins is sufficient to create the opaque *o2 *phenotype [46]. Although further research is needed to provide direct evidence of these relationships, the up-regulation of these genes is a strong indicator of the deleterious nature of the opaque mutations and their perturbation of endosperm cell functions.

The regulation of gene expression is central to a myriad of biological processes at the molecular level and is mainly controlled by transcription factors and signal transducers. These are of special interest since they are capable of coordinating the expression of several downstream target genes active in metabolic and developmental pathways and may provide new perspectives for data mining and for studies of coordinated gene regulation in developing maize endosperm. Additionally, TFs might be a powerful tool for the modification of metabolism and hence the generation of crops having superior characteristics because a single TF frequently regulates coordinated expression of a set of key genes involved in metabolic pathways. Although different regulatory mechanisms involving *O2 *have been suggested earlier on the basis of protein-protein interactions, we have identified, in addition to *O2*, other TFs that may be useful for clarifying the interaction between *O2 *and other putative TFs, such as MAD-box, Myc, and YABBY [9,46-49]. This last small plant-specific TF family contain TF family contains seven to eight members in rice and six in Arabidopsis, where they were shown to be involved in establishing abaxial-adaxial polarity in lateral organs and in restricting meristem nutrition and growth [50]. Characterization of these genes in monocots is less advanced, but mutational and expression analysis suggest that their functions have diverged between monocots and dicots, with the monocot TFs lacking a central role in specifying abaxial-adaxial cell fate [50]. They may represent candidates for genes primarily or secondarily involved in the control of metabolic networks, and their analysis can help to elucidate endosperm metabolism. Furthermore, *O2 *is able to recruit the maize co-activators GCN5 and ADA2 to modulate transcription of chimerical genes, showing that *O2 *is able to interact with proteins other than the bZIP type in heterologous systems. It is worth to mention that in the current study we have observed a sizeable reduction in the *o7 *endosperm of the transcription level of *O2 *and *VSF1 *(*vascular specificity factor 1*), another bZIP transcriptional activator, identified in tomato and involved in vascular development [51,52]. Whether *O7 *affects directly or indirectly expression of other TFs remains to be clarified. However, it is clear that the down-regulation of *O2 *noted in the *o7 *mutant is not sufficient to induce an *o2*-like phenotype, because changes in the transcriptome of the two mutants are different and appear to some extent additive. Therefore, it is likely that *O7 *is one of the components that may cooperate with other factor(s) in regulating *O2 *expression via direct or indirect mechanisms. Finally, our results indicate alterations in the expression profile of genes encoding protein phosphatases and kinases; these proteins, in turn, provide the means to transduce internal (e.g., hormones) and external (e.g. temperature) signals into transcriptional and/or chemical responses in cells. Almost no protein phosphatases and kinases from seeds have been analyzed in the opaque mutants in detail, although evidence has shown that at the post-translational level phosphorylation of O2 protein modulates its DNA-binding affinity [53]. In fact, these last authors have found that O2 proteins exist in the endosperm cells as a pool of differentially phosphorylated forms varying in their relative abundance and in the extent of phosphorylation.

## Conclusions

In summary, our analyses reveal that *O2 *and *O7 *are very pleiotropic regulatory factors, affecting the expression of a broad range of endosperm-expressed genes involved in several metabolic pathways. Here, the use of microarrays based on cDNA libraries of biological samples enriched in endosperm tissue allowed us to identify with a good level of confidence a large collection of genes differentially expressed in endosperm mutants that were not previously identified through traditional analyses and in a similar study as reported previously [7]. The number of genes to be affected by *O2 *and *O7 *suggests that these, and in particular *O2*, represent an evolutionary ancient factor responsible for modeling intermediary metabolism, which has been subsequently recruited for boosting the expression of α-zeins storage products. Although, by necessity this paper is descriptive and more work is necessary to define gene function and dissect the complex regulation of gene expression, the genes isolated and characterized to date give us an intriguing insight into the mechanisms underlying endosperm metabolism.

## Methods

### Plant material

The normal maize inbred A69Y(wt) and the endosperm mutant genotypes *o2*, *o7*, and *o2o7*, in a near-isogenic A69Y background were grown in adjacent plots in the genetic nursery of the Maize Research Unit in Bergamo (Italy), during summer 2006. The *o2 *mutant line contained *o2m*(*r*), a null expression *O2 *allele, while the *o7 *mutant was obtained from the Maize Genetics Stock Centre at the University of Illinois (Urbana-Champaign) [54]. All endosperm mutants' genotypes were converted to the A69Y background through six backcrossing cycles, following by several rounds of self-pollination; they are phenotypically uniform and appear genetically homogeneous as expected, because after six backcross generations the mutant inbred lines should share, on average, ~99% of the recurrent parent genome. The homozygous *o2o7 *double mutant was obtained by crossing the above-mentioned *o2 *and *o7 *A69Y lines, and selecting for the homozygous double mutant kernels.

A minimum of 8 well-filled ears for each genotype were sampled at 14 days after pollination (DAP), a stage where storage protein and starch syntheses commence, and frozen immediately in liquid nitrogen. Kernels were taken from the centre of each ear; the endosperm was dissected from the embryo and pericarp and stored at -80°C.

Mature kernels were harvested after physiological maturity and dried in a forced-air oven. To minimize the effect of biological variation between ears on gene expression, equal numbers of dissected endosperms from 4 ears were pooled and treated as one sample; thus a minimum of three replicated samples was used for each experiment.

### Total Nitrogen, protein and amino acid analysis

Protein analyses were performed with endosperm from mature kernels. Samples were freeze-dried, ground in a mortar, and analyzed for total nitrogen (N) content on an automated N analyzer (NA1500, Carlo Erba) following the method of Dumas. Total endosperm proteins were extracted in duplicate, from 10-20 endosperms and fractionated as previously described by [55]. The percentage of total protein (N*6.25) was calculated by subtracting the value of non-protein N evaluated from the value obtained for total N content [56].

Amino acids analysis (after Performic acid oxidation) was performed at the analytical facility of the University of Milan (Italy). Measurements were made with pooled samples of 15 kernels for each genotype; the data presented are the means of four independent assays.

### 2-D SDS-PAGE

Isoelectric focusing (IEF) was performed with a Multiphor II System (Pharmacia LKB Biotechnology AB, Uppsala, Sweden). 0.5 mm thick IEF gels containing 3.3% acrylamide/bis (A/B 28.8% AC 1.2% bis), 0.04% ammonium persulfate, 0.07% TEMED, Ampholine carrier ampholytes (Sigma, Dorset, UK): pH 3.5-10 (2.96%); pH 4-6 (0.52%); pH 5-7 (0.52%); pH 7-9 (0.52%); pH 8-10.5 (0.52%), and 6 M urea, were cast onto a gel-support medium (gel Bond PAG Film/FML Bio Products). Electrodes were placed at a distance of 13 cm. Wicks were soaked in 0.5 M H_3_PO_4 _(+) and 0.5 M NaOH (-). Sample wells (silicon 7 mm × 1 mm) were placed 1 cm from the anode and loaded with protein samples dissolved in IEF resuspension buffer (6 M urea, 50 mM K_2_CO_3_, 2% v/v 2-mercaptoethanol) [57] and with 10 μl pI markers (IEF mix 3.5-9.3, Sigma). IEF was performed at 8 W for 2 h. After IEF separation, one gel strip per well was cut out and equilibrated for 30 min. in 1.12 M glycerol, 75mM Tris-HCl pH 6.8, 2.4% (v/v) SDS and 2.5% 2-mercaptoethanol. For the second dimension, a 15% Laemmli gel with a 2 cm stacking gel was cast without slot former and the IEF strip was then mounted at the cathodic end. After SDS-PAGE, gels were stained and dried.

### cDNA cloning and microarray construction

Microarrays were assembled using clones obtained during the EC "Zeastar" project (http://www.cerealsdb.uk.net/zeastar.htm). Briefly, 20 part-normalized cDNA libraries were prepared from 3-28 DAP endosperm and kernel development tissues covering the 5 key stages (i.e. cenocytic, cellularization, differentiation, reserve synthesis, and maturation [2]. 22,365 ESTs were sequenced, aligned, assembled into contigs using a similarity score of 90%, and annotated using BLASTX (96% id/75 bp) software. For each contig, the cDNA containing the largest transcript was identified. These, together with all singleton cDNAs (6719) were used to construct a Unigene set of 8,950 sequences. ESTs were stored as cloned fragments in glycerol stocks in 384-well microtiter plates at -80°C. Before spotting, 2 μl of each EST sample were added to 50 μl PCR amplifications using: 2 μl of T3 primer at 15 pmol/μl; 2 μl of T7 primer at 15 pmol/μl; 5 μl of 2 mM dNTP mix; 1.5 μl of 50 mM MgCl_2_; 5 μl of Invitrogen 10x PCR reaction buffer; 0.2 μl of Invitrogen Taq DNA polymerase recombinant (5 U/μl). Amplified products were purified with the Wizard MagneSil PCR Clean Up System (Promega, Madison, WI). Aliquots were then tested on 0.8% agarose gels in order to verify insert integrity and concentration. Finally, selected amplification products were air-dried and resuspended in 15 μl of 3x printing buffer (150 mM of NaH_2_PO_4_, 150 mM Na_2_HPO_4_, pH 8.5).

### mRNA isolation and slide hybridization

Total RNA was prepared from 100g frozen, ground endosperm tissue using Trizol Reagent (Invitrogen, Carlsbad, CA) following the manufacturer's instructions. polyA^+ ^RNA was purified using the PolyA Tract mRNA System Kit IV (Promega, Madison, WI) following two cycles of oligo(dT) column purification to ensure a high purity of polyA^+ ^RNA. The purified RNA was quantified by measuring its absorbance at 260 nm and diluted to a final concentration of 1 μg/μl.

For each mRNA probe, 1 μg of purified polyA^+ ^RNA was labelled by reverse transcription in the presence of Cy3- and Cy5- dCTP using the Amersham CyScribe-First Strand cDNA Labelling kit (Amersham Biosciences, Piscataway, NJ) following manufacturer's indications. Microarray slides were placed in a rack and incubated as follows: 1) 15-30 min at 50°C in pre-warmed pre-hybridization solution 1 (3x SSC, 0.1% SDS 0.1 mg/ml BSA); 2) two rapid washes in distilled water; 3) 20-40 min at 50°C in pre-warmed pre-hybridization solution 2 (4x SSC, 0.1% SDS); 4) 2 min in distilled water at 94°C for sample denaturation; 5) two washes at RT in distilled water. Subsequently, slides were centrifuged at 1,500 rpm for 3 min at RT. Labelled cDNA mixes (Cy3 and Cy5) were added to 15 μl of formamide and 7.5 μl of Amersham 5x microarray hybridization buffer (Amersham Biosciences, Piscataway, NJ). The mixture was dispensed onto the microarray slides, covered with a Hybri-Slip cover slip (Sigma, Dorset, UK) and incubated in the dark at 42°C overnight in a hybridization chamber (GeneMachines, San Carlos, CA) containing 120 μl of sterile distilled water to maintain humidity. Hybridized slides were washed as follows: 1) 5 min at 42°C with 2x SSC, 0.1% SDS; 2) 5 min at 42°C with 1x SSC, 0.1% SDS; 3) 5 min at RT in 0.2x SSC; 4) 5 min at RT in 0.1x SSC; 5) 5 min at RT in distilled water. Finally, the slides were centrifuged at 1,500 rpm for 3 min to remove remaining liquid.

### Microarray data analysis

All microarray experiments were performed in triplicate using dye swapping, hence giving rise to 12 independent measurements for each EST, considering the presence of duplicate spots on each slide. Raw measurements of spot fluorescence intensities were collected from hybridized slides using a Genepix 4100A scanner and Genepix Pro4 software (Axon Instruments, Union, CA). Subsequently and with the use of the TM4 software suite [58], the obtained spot values were corrected for background fluorescence and inconsistent hybridization results across dye-swap replicates. The data were log_2 _transformed and LOWESS normalized correcting for pin-induced spot intensity biases. To verify reproducibility between spots across slides, F-tests were performed applying a 95% confidence threshold and allowing removal of inconsistent hybridization results. A mixed model ANOVA was used to assess the significance of the difference in expression of each gene among genotypes using a false-discovery rate significance threshold of 0.05 [59,60]. With the multiple steps required to carry out a successful microarray experiment, it is not unusual to have "noisy" data. To extract reliable information from the data, non-biologically significant sources of signal variation were identified and their effects removed. The following gene model was used to identify genes that were differentially expressed:

*Y*_*ijkl *_denotes the transformed intensity for a gene, *μ *denotes the average intensity and *ε*_*ijklm *_captures the random errors. The variation due to microarray slide used (Array) was designated as random effect, whereas, variations due to RNA fluorescent labeling (Dye), biological sample RNA (BioRep) and endosperm genotype (Treatment) were treated as fixed effects. Only the main effects interacting with Treatment were included in the model.

### Quantitative Real-Time PCR

1 μg of mRNA was reverse transcribed by mixing with 1 μl of oligo-dT_18 _(2 μg/μl), 1 μl of dNTP mix (10 mM), 4 μl of first strand buffer (Invitrogen, Carlsbad, CA), 2 μl of 0.1 M DTT, 1 μl (200 U/μl) of M-MLV Reverse Transcriptase (Invitrogen, Carlsbad, CA), and 13 μl of distilled sterile water. After reverse transcription at 37°C for 1 hour, the cDNAs were tested on a 0.8% agarose gel and diluted to a final volume of 500 μl with distilled sterile water. PCR reaction mixtures were assembled combining: 2 μl of diluted cDNA; 2 μl of gene-specific forward primer (15 pmol/μl), 2 μl of gene-specific reverse primer (15 pmol/μl), 5 μl of 10x reaction buffer (Invitrogen, Carlsbad, CA), 2 μl of 50 mM MgCl_2_, 2.5 μl of 2 mM dNTP mix, 5 μl of diluted (5,000 fold) SYBR Green, 0.5 fluorescein, 0.2 μl of Platinum Taq DNA polymerase (5 U/μl, Invitrogen, Carlsbad, CA). Real-Time amplification was performed using an iCycler (BioRad, Hercules, CA) using the following thermal cycling profile: 95°C for 5 min followed by 50 cycles of 95°C - 30 sec; 55°C - 30 sec; 72°C - 30 sec. All reactions were performed in triplicate. The obtained threshold cycles (C_T_) were averaged across replicates and sample errors computed. Ratios of C_T _values were computed and used to corroborate the observed hybridization patterns. Linear regression analyses showed a strong correlation between measurements of gene expression assessed by microarrays and by qRT-PCR, with correlation coefficient r^2 ^= 0.83 (n= 120, r = 0.91; data not shown). Gene-specific primers were selected and designed from sequences near the 3' end of the gene using the Zeastar Unigene sequence database. An 18S rRNA was selected as a control.

### Sequence confirmation of clones

To confirm the fidelity of differentially expressed genes, corresponding clones were sequenced from the 5' end using a universal reverse primer on an automatic DNA sequencer (CEQ 8000, Beckman-Coulter, Fullerton, CA).

## Authors' contributions

HH and MM designed the study and drafted the manuscript. ML carried out amino acid and protein related analyses, NL and RP carried out the microarray analysis and general molecular work. HH performed the qRT-PCR and collected and analyzed the data. All authors read and approved the final version.

## Supplementary Material

Additional file 1 **Table S1 - differently expressed genes identified**. Complete list of genes whose mRNA levels differed significantly (P > 0.05) between wild-type (WT) and opaque (*o2*, *o7*, *o2o7*) endosperm mutants as identified in this paper. Click here for file
