# Supplementary material for: The Zea mays mutants opaque-2 and opaque-7 disclose extensive changes in endosperm metabolism as revealed by protein, amino acid, and transcriptome-wide analyses
Source: BMC Genomics. 2011 Jan 18;12:41. doi: 10.1186/1471-2164-12-41 (PMC3033817; doi:10.1186/1471-2164-12-41)
Supplement: Additional file 1 — Table S1 - differently expressed genes identified. Complete list of genes whose mRNA levels differed significantly (P > 0.05) between wild-type (WT) and opaque (o2, o7, o2o7) endosperm mutants as identified in this paper. [file 1471-2164-12-41-S1.DOC]

| Contig | WT/o2 ratio | WT/o7 ratio | WT/o2o7 ratio | WT fluo signal | Functional class | Description | Homology |
| --- | --- | --- | --- | --- | --- | --- | --- |
| Zeastar-A01-B03 |  | 5.5 |  | 30496.88 | nucleotide metabolism | putative phosphoribosyl pyrophosphate synthase | EAZ13389.1 |
| Zeastar-A01-E04 | 2.3 | 1.8 |  | 8640.63 | translation | ribosomal protein L32 | EAZ09640.1 |
| Zeastar-A01-E05 | 1.7 |  |  | 10096.88 | transcription | putative transcription factor BTF3 | EAZ25311.1 |
| Zeastar-A01-E12 |  |  | 0.4 | 13187.5 | protein folding | 10 kDa chaperonin | AAB63591.1 |
| Zeastar-A01-F06 | 2.1 |  |  | 3568.75 | transport | vacuolar ATPase - β subunit | EAZ37403.1 |
| Zeastar-A01-H06 | 2.5 |  |  | 3996.88 | transport | transport protein | EAZ18241.1 |
| Zeastar-A02-C02 | 1.8 | 1.7 |  | 31481.25 | cell wall | endo-1.4-β-glucanase | ABF98747.1 |
| Zeastar-A02-E09 | 1.8 |  |  | 7512.5 | cytoskeleton | actin | EAZ32588.1 |
| Zeastar-A03-B02 | 2.4 |  |  | 253.13 | cytoskeleton | α-tubulin | P14641.1 |
| Zeastar-A03-B07 | 1.8 | 1.6 |  | 38356.25 | cytoskeleton | actin | EAY96234.1 |
| Zeastar-A03-F05 |  | 1.8 |  | 2768.75 | signal transduction | protein phosphatase 2A | NP_001105904.1 |
| Zeastar-A03-G04 | 2.2 |  |  | 6175 | signal transduction | GTP-binding protein - YPTM3 | NP_001105743.1 |
| Zeastar-A04-F12 |  | 1.6 |  | 3925 | nucleotide metabolism | putative small nuclear ribonucleoprotein polypeptide | NP_001050345.1 |
| Zeastar-A05-C03 | 2.4 |  |  | 6937.5 | carbohydrate metabolism | 6-phosphogluconolactonase-like protein (**EC 3.1.1.31**) | NP_001060218.1 |
| Zeastar-A05-E05 |  | 1.6 |  | 8287.5 | protein turnover | 26S proteasome regulatory particle | NP_001059500.1 |
| Zeastar-A05-F03 | 2.1 |  |  | 18021.88 | signal transduction | small RAS-related GTP-binding protein | CAO65615.1 |
| Zeastar-A05-H02 | 2.9 |  |  | 2450 | xenobiotic biodegradation | putative carboxymethylenebutenolidase | EAZ12194.1 |
| Zeastar-A05-H03 | 3.2 |  |  | 13087.5 | xenobiotic biodegradation | putative carboxymethylenebutenolidase | EAZ12194.1 |
| Zeastar-A06-B10 | 2.3 |  |  | 2903.13 | electron transport | putative cytochrome B5 | NP_001045534.1 |
| Zeastar-A06-G08 | 2.8 | 1.6 |  | 11612.5 | translation | ribosomal protein L1 | EAZ43641.1 |
| Zeastar-A06-H11 |  | 1.7 |  | 18615.63 | translation | translation initiation factor | AAD20980.1 |
| Zeastar-A07-G12 | 4.6 | 1.8 |  | 3646.88 | transcription | histone deacetylase 2 | Q9M4U4 |
| Zeastar-A07-H05 | 3 |  |  | 29953.13 | translation | 60S ribosomal protein L17 | NP_001048495.1 |
| Zeastar-A07-H09 |  | 4.6 |  | 6900 | signal transduction | protein kinase | NP_001105555.1 |
| Zeastar-A08-B05 | 1.9 |  |  | 3796.88 | AA metabolism | methionine synthase protein (**EC 2.1.1.14**) | AAL73979.1 |
| Zeastar-A08-C01 | 2.8 |  |  | 11087.5 | translation | 40S ribosomal subunit protein S21 | NP_001105477.1 |
| Zeastar-A08-D01 | 3.3 | 1.9 |  | 7090.63 | translation | elongation factor 1 | EAZ40650.1 |
| Zeastar-A08-E09 | 2.3 |  |  | 475193.75 | stress - defense response | heat shock factor-binding protein | NP_001105090.1 |
| Zeastar-A08-H03 |  | 1.8 |  | 2396.88 | protein turnover | proteasome component | NP_001047516.1 |
| Zeastar-A08-H08 | 2.3 | 1.6 |  | 7762.5 | signal transduction | putative auxin-induced protein | EAY74981.1 |
| Zeastar-A09-C01 | 2.5 |  |  | 5281.25 | carbohydrate metabolism | putative malate dehydrogenase (**EC 1.1.1.37**) | NP_001056389.1 |
| Zeastar-A09-G06 |  | 1.7 |  | 10925 | translation | putative methionine aminopeptidase | ABB47866.1 |
| Zeastar-A09-G08 | 3.5 |  |  | 22106.25 | molecular chaperonine | seed maturation protein PM37 | EAY92107.1 |
| Zeastar-A09-G12 | 2.6 |  |  | 6115.63 | cytoskeleton | α-tubulin | P14641.1 |
| Zeastar-A09-H01 | 1.8 | 1.7 |  | 18500 | translation | putative 40S ribosomal protein S19 | NP_001050397.1 |
| Zeastar-A10-A02 | 2.3 | 1.6 |  | 47050 | stress - defense response | heat shock protein 86 | NP_001062159.1 |
| Zeastar-A10-A06 | 3 | 1.8 |  | 6821.88 | translation | ribosomal protein L38 | NP_001067801.1 |
| Zeastar-A10-A07 | 2.5 |  |  | 1603.13 | translation | putative 60S ribosomal protein L25 | EAY94740.1 |
| Zeastar-A10-B05 | 1.8 | 1.7 |  | 290843.75 | translation | translation initiation factor 5a | EAZ04583.1 |
| Zeastar-A10-C03 | 2.1 |  |  | 2228.13 | protein turnover | ubiquitin-protein ligase 2 | EAZ20314.1 |
| Zeastar-A10-D06 | 1.9 |  |  | 6009.38 | sterol metabolism | putative oxysterol-binding protein | EAZ26431.1 |
| Zeastar-A10-D09 | 2.2 |  |  | 112425 | carbohydrate metabolism | sucrose synthase (**EC 2.4.1.13**) | NP_001105194.1 |
| Zeastar-A10-G05 | 0.4 |  |  | 71909.38 | stress - defense response | putative low temperature and salt responsive protein | EAY96485.1 |
| Zeastar-A10-G07 |  | 1.8 |  | 3715.63 | splicing | splicing factor-like protein | NP_001064686.1 |
| Zeastar-A10-H12 | 2 | 1.6 |  | 4878.13 | cytoskeleton | α-tubulin | P14641.1 |
| Zeastar-B01-A01 |  | 1.8 |  | 255384.38 | signal transduction | putative Ser/Thr specific protein | EAZ12582.1 |
| Zeastar-B01-C03 | 2.9 | 1.6 |  | 19418.75 | molecular chaperonine | putative T-complex protein 1 | NP_001058409.1 |
| Zeastar-B01-C04 | 2.2 |  |  | 14171.88 | protein turnover | β1 subunit of 20S proteasome | NP_001056748.1 |
| Zeastar-B01-D04 | 2.6 | 1.6 |  | 21443.75 | translation | putative elongation factor | NP_001046972.1 |
| Zeastar-B01-F05 |  | 1.6 |  | 13225 | electron transport | cytochrome B5 | NP_001054434.1 |
| Zeastar-B01-G12 | 0.5 |  |  | 11993.75 | transcription factor | MADS box protein | NP_001105332.1 |
| Zeastar-B03-B02 | 2.4 | 1.7 |  | 127000 | membrane | membrane intrinsic protein | NP_001105022.1 |
| Zeastar-B03-C08 | 2.2 |  |  | 4546.88 | AA metabolism | putative ketol-acid reductoisomerase (**EC 1.1.1.86**) | ABR25710.1 |
| Zeastar-B03-G06 | 1.9 |  | 2.1 | 26675 | carbohydrate metabolism | succinate dehydrogenase subunit (**EC 1.3.5.1**) | EAZ38613.1 |
| Zeastar-B03-H03 | 1.8 | 1.7 |  | 3346.88 | signal transduction | calmodulin | NP_001055907.1 |
| Zeastar-B03-H07 |  | 1.8 |  | 9215.63 | butanoate metabolism | hydroxymethylglutaryl coenzymeA synthase | EAZ25374.1 |
| Zeastar-B04-A04 | 1.8 | 1.6 |  | 13887.5 | stress - defense response | LRRR protein | EAZ13889.1 |
| Zeastar-B04-D01 |  | 1.6 |  | 2606.25 | transport | voltage-dependent anion channel | NP_001105453.1 |
| Zeastar-B04-E08 | 2.2 | 1.9 |  | 5090.63 | translation | ribosomal protein S7 | NP_001049835.1 |
| Zeastar-B04-H05 |  | 1.8 |  | 27418.75 | signal transduction | Ser/Thr-protein kinase | ABU88852.1 |
| Zeastar-B04-H08 |  | 1.8 |  | 15234.38 | signal transduction | Ser/Thr-protein kinase | NP_001105403.1 |
| Zeastar-B04-H10 | 2.4 |  |  | 7134.38 | energy metabolism | cytochrome C oxidase subunit (**EC 1.9.3.1**) | Q42841 |
| Zeastar-B05-C07 | 3.1 | 1.7 |  | 25081.25 | electron transport | putative cytochrome B5 | NP_001045534.1 |
| Zeastar-B05-D07 | 1.8 |  |  | 268331.25 | cytoskeleton | dynamin homolog | NP_001057285.1 |
| Zeastar-B05-G11 | 1.9 |  |  | 11003.13 | transport | putative α-soluble NSF attachment protein | NP_001061446.1 |
| Zeastar-B06-D02 |  | 2.1 |  | 16109.38 | protein turnover | nucellin-like protein | AAM76716.1 |
| Zeastar-B06-E05 | 1.9 |  |  | 3750 | translation | 40S ribosomal protein S27 homolog | NP_001105614.1 |
| Zeastar-B07-B10 | 2.1 | 2 |  | 11090.63 | energy metabolism | methylenetetrahydrofolate reductase (**EC 1.5.1.20**) | NP_001104947.1 |
| Zeastar-B07-D06 | 2.5 | 1.9 |  | 5809.38 | signal transduction | shaggy-like kinase | EAY85137.1 |
| Zeastar-B07-D09 | 3.2 | 10 |  | 2640.63 | cytoskeleton | kinesin-like protein | EAZ08128.1 |
| Zeastar-B08-A08 | 3.5 | 1.8 |  | 2165.63 | translation | putative 40S ribosomal protein | ABF97261.1 |
| Zeastar-B08-C05 | 2.3 | 2.9 |  | 73040.63 | carbohydrate metabolism | malate dehydrogenase (**EC 1.1.1.37**) | AAK58078.1 |
| Zeastar-B08-D09 | 1.8 | 1.8 |  | 114159.38 | nucleotide metabolism | putative small nuclear ribonucleoprotein U1A | EAZ32930.1 |
| Zeastar-B08-F10 | 2.3 | 2 |  | 5628.13 | carbohydrate metabolism | D-TDP-glucose dehydratase (**EC 4.2.1.46**) | CAC14890.1 |
| Zeastar-B08-H12 | 2.4 |  | 0.5 | 10384.38 | protein turnover | putative pre-pro-cysteine proteinase | CAA71892.1 |
| Zeastar-B09-B09 | 1.7 |  |  | 21346.88 | translation | putative translation initiation factor SUI1 | EAZ04141.1 |
| Zeastar-B09-D04 | 2 | 1.6 |  | 91009.38 | AA metabolism | serine hydroxymethyltransferase (**EC 2.1.2.1**) | EAZ18268.1 |
| Zeastar-B09-D10 | 2.7 | 1.8 |  | 10240.63 | protein folding | cyclophilin isomerase | EAZ38239.1 |
| Zeastar-B09-G03 | 2 |  |  | 11068.75 | RNA modification | serine/arginine-rich protein - SC35-like splicing factor | EAZ04813.1 |
| Zeastar-B09-H01 |  | 4.5 |  | 3268.75 | AA metabolism | putative glutathione synthetase (**EC 6.3.2.3**) | NP_001068356.1 |
| Zeastar-B09-H02 | 1.9 |  |  | 3453.13 | translation | ribosomal protein L32 | EAZ09640.1 |
| Zeastar-B10-A08 | 2.4 | 1.7 |  | 3009.38 | nucleotide metabolism | adenosine kinase | CAB40376.1 |
| Zeastar-B10-B05 | 2.5 | 1.8 |  | 7700 | energy metabolism | pyrophosphatase (**EC 3.6.1.1**) | AAA61610.1 |
| Zeastar-B10-C09 | 2.1 |  | 0.5 | 15815.63 | translation | eukaryotic translation initiation factor 5 | CAA10616.1 |
| Zeastar-B10-D03 |  | 3.3 |  | 5906.25 | transcription | SSRP1 protein | NP_001105124.1 |
| Zeastar-B10-E06 | 2.1 | 1.6 |  | 273340.63 | translation | ribosomal protein L32 | EAZ09640.1 |
| Zeastar-B10-G07 | 2.6 |  |  | 7312.5 | protein turnover | putative disulfide isomerase | NP_001105759.1 |
| Zeastar-C01-H02 |  |  | 0.4 | 19940.63 | cytoskeleton | annexin P35 | NP_001105475.1 |
| Zeastar-C02-B08 | 1.8 |  |  | 4212.5 | signal transduction | map kinase MAPK2 | ABC02871.1 |
| Zeastar-C02-C06 |  | 1.9 |  | 3409.38 | carbohydrate metabolism | uridine diphosphate glucose epimerase | EAZ06795.1 |
| Zeastar-C02-D04 | 2.1 | 1.7 |  | 9756.25 | transport | putative ADP-ribosylation factor | BAB90396.1 |
| Zeastar-C02-F07 |  | 1.6 |  | 6628.13 | structural | coated vesicle membrane protein | EAY91831.1 |
| Zeastar-C02-H05 | 1.9 |  |  | 7703.13 | translation | ribosomal protein L32 | EAZ09640.1 |
| Zeastar-C03-A03 | 2.4 |  |  | 15218.75 | cytoskeleton | delta-cop (coatomer delta subunit) | NP_001104961.1 |
| Zeastar-C03-B05 |  | 2.5 |  | 5115.63 | carbohydrate metabolism | granule binding starch synthase II (**EC 2.4.1.11**) | NP_001106039.1 |
| Zeastar-C03-F11 | 2.4 | 1.6 |  | 8690.63 | translation | ribosomal protein S29 | NP_001068325.1 |
| Zeastar-C04-A03 | 2.3 |  |  | 85121.88 | translation | elongation factor 1 β2 | EAZ40650.1 |
| Zeastar-C04-D02 |  | 1.6 |  | 303956.25 | energy metabolism | NADPH-cytochrome P450 reductase (**EC 1.6.2.4**) | CAE01547.2 |
| Zeastar-C04-D08 |  | 2 |  | 18746.88 | transcription regulator | MADS box protein | CAB85962.1 |
| Zeastar-C05-B06 | 2 |  |  | 37040.63 | protein turnover | putative ubiquitin-conjugating enzyme | CAO15839.1 |
| Zeastar-C05-E04 |  | 1.7 |  | 3950 | cell wall | cellulose synthase-3 | AAF89963.1 |
| Zeastar-C05-E12 | 2.4 | 1.7 |  | 4168.75 | molecular chaperonin | peptidylprolyl isomerase | NP_001062292.1 |
| Zeastar-C05-H08 | 1.8 |  |  | 36393.75 | translation | putative 40S ribosomal protein S5 | NP_001067900.1 |
| Zeastar-C06-G10 | 1.9 |  |  | 7662.5 | translation | cytoplasmic ribosomal protein L18 | NP_001054688.1 |
| Zeastar-C06-H03 | 2.6 |  |  | 74853.13 | protein turnover | 20S proteasome subunit α | NP_001068278.1 |
| Zeastar-C07-E02 | 1.9 |  | 0.5 | 60843.75 | stress - defense response | HSP70 | EAY82726.1 |
| Zeastar-C07-G01 | 1.9 |  |  | 3671.88 | cytoskeleton | β-tubulin 2 | CAO42578.1 |
| Zeastar-C07-G07 |  | 1.7 | 2.9 | 33793.75 | RNA modification | putative splicing factor | EAY88585.1 |
| Zeastar-C07-G08 | 1.8 |  |  | 3815.63 | translation | putative eukaryotic translation initiation factor 3 | NP_001105726.1 |
| Zeastar-C08-D07 | 2.6 |  |  | 8112.5 | energy metabolism | putative nucleoside diphosphate kinase (**EC 2.7.4.6**) | P93554 |
| Zeastar-C08-H09 | 4 |  |  | 103809.38 | fatty acid metabolism | putative stearoyl-acyl-carrier protein desaturase (**EC 1.14.19.2**) | ABA40393.1 |
| Zeastar-C09-A07 | 3.2 | 1.7 |  | 33468.75 | nucleotide metabolism | putative small nuclear ribonucleoprotein | ABK93602.1 |
| Zeastar-C09-C04 | 3.7 | 2 |  | 400456.25 | translation | translation initiation factor 4A2 | NP_001045878.1 |
| Zeastar-C09-C10 |  | 1.9 |  | 5878.13 | translation | ribosomal protein L17-1 | NP_001104890.1 |
| Zeastar-C09-C12 | 1.9 |  |  | 6050 | signal transduction | cysteine proteinase precursor | NP_001105685.1 |
| Zeastar-C09-D04 | 2.4 |  |  | 24040.63 | AA metabolism | cysteine synthase (**EC 2.5.1.47**) | NP_001105469.1 |
| Zeastar-C09-D05 | 3.7 | 1.6 |  | 2806.25 | AA transport | amino acid transporter-like protein | EAZ37724.1 |
| Zeastar-C09-D10 |  | 1.8 |  | 2478.13 | mRNA processing | mRNA capping enzyme | EAY80377.1 |
| Zeastar-C10-E02 |  | 1.8 |  | 18765.63 | AA metabolism | 2-dehydro-3-deoxyphosphoheptonate aldolase precursor (**EC 2.5.1.45**) | EAZ04751.1 |
| Zeastar-C10-F11 | 2.3 |  |  | 10778.13 | replication and repair | RAD6 | EAY96797.1 |
| Zeastar-E01-G02 | 3.1 | 1.7 |  | 6784.38 | translation | acidic ribosomal protein P2A | NP_001105388.1 |
| Zeastar-E01-G12 | 1.8 |  |  | 8237.5 | transport | zinc transporter | CAL18286.1 |
| Zeastar-E01-H04 | 2.1 |  |  | 28731.25 | energy metabolism | thioredoxin (**EC 1.8.1.9**) | NP_001105788.1 |
| Zeastar-E01-H07 | 2 | 1.6 |  | 17575 | cell wall | cellulose synthase-2 | NP_001105574.1 |
| Zeastar-E02-A01 |  |  | 0.4 | 3753.13 | RNA modification | putative polyA-binding protein | EAZ31059.1 |
| Zeastar-E02-A12 | 2.7 | 1.7 |  | 4793.75 | signal transduction | learca2 protein | NP_001043910.1 |
| Zeastar-E02-D05 | 1.8 |  |  | 8331.25 | translation | putative elongation factor 1b | EAY85044.1 |
| Zeastar-E02-D08 | 2.2 |  |  | 9140.63 | translation | ribosomal protein S6 | NP_001105544.1 |
| Zeastar-E02-D10 | 1.9 |  |  | 6665.63 | carbohydrate metabolism | NAP-dependent isocitrate dehydrogenase precursor (**EC 1.1.1.41**) | NP_001053093.1 |
| Zeastar-E02-E08 | 2 |  |  | 27168.75 | fatty acid metabolism | squalene epoxidase-like protein (**EC 1.14.99.7**) | NP_001049463.1 |
| Zeastar-E02-E12 | 1.9 |  |  | 14071.88 | carbohydrate metabolism | phosphoglucomutase (**EC 5.4.2.2**) | NP_001105703.1 |
| Zeastar-E02-F08 | 2.3 | 1.9 | 44.9 | 5571.88 | translation | 60S ribosomal protein L12-like | EAY95398.1 |
| Zeastar-E02-F12 |  |  | 0.3 | 7915.63 | carbohydrate metabolism | phosphoglycerate kinase (**EC 2.7.2.3**) | EAZ34803.1 |
| Zeastar-E02-H05 | 2.5 |  |  | 2353.13 | signal transduction | calmodulin | NP_001042688.1 |
| Zeastar-E02-H06 |  | 1.6 |  | 4709.38 | nucleotide metabolism | putative small ribonucleoprotein | EAY78995.1 |
| Zeastar-E03-A05 | 3.6 | 2.1 | 2 | 4487.5 | signal transduction | activated protein kinase c receptor homolog | ABR25943.1 |
| Zeastar-E03-C03 | 1.7 |  |  | 1668.75 | transcription | putative transcription factor BTF3 | NP_001051911.1 |
| Zeastar-E03-C10 | 2.3 |  |  | 15246.88 | translation | elongation factor | AAF99703.1 |
| Zeastar-E03-D03 | 2.1 |  |  | 4531.25 | stress - defense response | putative cold-induced protein | NP_001056613.1 |
| Zeastar-E03-E02 |  | 1.7 |  | 3012.5 | signal transduction | plasma membrane H+ ATPase | EAZ21357.1 |
| Zeastar-E03-F04 | 2.6 |  |  | 6112.5 | DNA folding | putative histone H2A | NP_001105357.1 |
| Zeastar-E03-G07 | 0.3 |  |  | 15800 | signal transduction | protein phosphatase | NP_001105341.1 |
| Zeastar-E03-H04 |  | 2.2 | 10.1 | 55631.25 | stress - defense response | putative purple acid phosphatase | NP_001049500.1 |
| Zeastar-E03-H05 |  | 1.6 | 4.9 | 3868.75 | replication and repair | RAD23 protein | EAZ00429.1 |
| Zeastar-E04-B03 |  | 1.7 | 2.4 | 18937.5 | cytoskeleton | putative tubulin β-4 chain | CAO67326.1 |
| Zeastar-E04-C05 | 1.7 |  |  | 4309.38 | signal transduction | adenylate kinase (**EC 2.7.4.3**) | NP_001067759.1 |
| Zeastar-E04-D06 |  | 1.9 | 10 | 23562.5 | cofactor - vitamin metabolism | putative thiamin biosynthesis protein | EAZ28134.1 |
| Zeastar-E04-F05 |  |  | 2.3 | 43712.5 | carbohydrate metabolism | putative pyruvate kinase (**EC 2.7.1.40**) | NP_001065454.1 |
| Zeastar-E04-G04 |  | 1.7 |  | 19609.38 | stress - defense response | LRR protein | NP_001044578.1 |
| Zeastar-E04-G08 | 0.3 |  |  | 4606.25 | carbohydrate metabolism | glycolate oxidase-like. (**EC 1.1.3.15**) | EAZ38786.1 |
| Zeastar-E04-G10 | 2.9 |  |  | 6609.38 | carbohydrate metabolism | ADP-glucose pyrophosphorylase large subunit (**EC 2.7.7.27**) | AAB24191.2 |
| Zeastar-E04-H05 | 2.7 | 1.7 |  | 12668.75 | carbohydrate metabolism | malate dehydrogenase (**EC 1.1.1.37**) | NP_001056389.1 |
| Zeastar-E04-H09 | 2.3 |  | 2.6 | 25015.63 | stress - defense response | HSP82 | EAZ43234.1 |
| Zeastar-E05-A04 | 1.8 |  |  | 8109.38 | cytoskeleton | α-tubulin | ABR25510.1 |
| Zeastar-E05-G02 |  | 1.7 |  | 3712.5 | translation | ribosomal protein S19 | BAC19887.2 |
| Zeastar-E06-A08 | 2 |  |  | 2925 | transport | phosphate - phosphoenolpyruvate translocator | AAB40650.1 |
| Zeastar-E06-A12 | 0.3 |  |  | 8796.88 | AA metabolism | glycine decarboxylase (**EC 1.4.4.2**) | AAB82711.1 |
| Zeastar-E06-C06 |  | 1.6 |  | 1575 | stress - defense response | HSP17.9 | CAA63903.1 |
| Zeastar-E06-D01 | 1.7 |  |  | 13071.88 | transcription | putative MADS-domain transcription factor | NP_001105525.1 |
| Zeastar-E06-E06 |  | 4.4 |  | 6121.88 | signal transduction | protein phosphatase 2A | A2X2K3.2 |
| Zeastar-E06-E07 |  | 1.9 | 38.1 | 2690.63 | translation | ribosomal protein L17-1 | NP_001104890.1 |
| Zeastar-E06-F01 |  |  | 0.4 | 8259.38 | stress - defense response | ABA- and ripening- induced protein | ABW06772.1 |
| Zeastar-E06-H06 |  | 1.7 |  | 3818.75 | signal transduction | putative RIC1 orysa RAS-related protein | NP_001043336.1 |
| Zeastar-E07-D01 |  | 1.7 |  | 11162.5 | carbohydrate metabolism | pectate lyase-like protein (EC 4.2.2.2) | EAY74463.1 |
| Zeastar-E07-D10 | 2 | 1.8 |  | 18796.88 | energy metabolism | putative ubiquinol-cytochrome C reductase (**EC 1.10.2.2**) | EAZ23360.1 |
| Zeastar-E07-D11 |  | 1.9 |  | 3912.5 | energy metabolism | NADH dehydrogenase-like (**EC 1.6.5.3**; **1.6.99.3**) | EAZ04506.1 |
| Zeastar-E07-F12 | 1.9 |  |  | 29406.25 | translation | translation initiation factor 5A | NP_001105606.1 |
| Zeastar-E07-G02 | 1.7 |  |  | 526878.13 | translation | 40S ribosomal protein S15 | P31674 |
| Zeastar-E08-A05 | 1.8 |  |  | 100871.88 | signal transduction | putative serine - threonine protein kinase | AAP50960.1 |
| Zeastar-E08-B08 | 2.1 |  |  | 8515.63 | translation | elongation factor 1α | NP_001105587.1 |
| Zeastar-E08-C09 | 3.2 | 1.7 |  | 65178.13 | DNA folding | putative histone H2A | NP_001105357.1 |
| Zeastar-E08-E05 |  |  | 67.2 | 15228.13 | stress - defense response | NTGP4 | EAY86287.1 |
| Zeastar-E09-A03 | 2.7 |  | 2.7 | 265659.38 | translation | putative elongation factor 1B-γ | EAY85044.1 |
| Zeastar-E09-A05 | 2.4 |  |  | 3068.75 | translation | putative elongation factor 1B-γ | ABR25394.1 |
| Zeastar-E09-A10 | 1.8 | 1.7 |  | 19493.75 | translation | 60S ribosomal protein L7 | EAZ41966.1 |
| Zeastar-E09-B12 | 2 |  |  | 31746.88 | carbohydrate metabolism | putative lipoamide dehydrogenase (**EC 1.8.1.4**) | EAY96621.1 |
| Zeastar-E09-C02 |  | 1.6 |  | 280415.63 | AA metabolism | caffeic acid 3-o-methyltransferase (**EC 2.1.1.6**) | AAQ24338.1 |
| Zeastar-E09-E06 | 2.7 |  |  | 18343.75 | translation | 40S ribosomal protein S15 | EAZ38933.1 |
| Zeastar-E09-E10 |  |  | 6.3 | 61540.63 | translation | putative ribosomal protein L28 | EAY88056.1 |
| Zeastar-E09-F05 | 1.8 |  |  | 4546.88 | energy metabolism | pyrophosphatase (**EC 3.6.1.1**) | NP_001054459.1 |
| Zeastar-E09-F07 |  | 1.6 | 4 | 59240.63 | transport | partial coat protein | CAA66847.1 |
| Zeastar-E09-G04 | 2.3 |  |  | 25431.25 | DNA folding | histone H3 | CAA31966.1 |
| Zeastar-E09-G08 | 1.8 |  |  | 3509.38 | transcription | DNA-directed RNA polymerase IIb | NP_001050570.1 |
| Zeastar-E09-H02 |  | 1.8 | 11.2 | 211825 | seed storage protein | 19 kDa α-zein | NP_001106012.1 |
| Zeastar-E10-A04 |  | 1.6 | 7.3 | 14406.25 | carbohydrate metabolism | putative aldose reductase (**EC 1.1.1.200**) | EAY76488.1 |
| Zeastar-E10-A05 | 0.5 |  | 8.6 | 3340.63 | protein turnover | 26S proteasome ATPase subunit RPT6 | ABR25526.1 |
| Zeastar-E10-A11 |  | 1.9 | 8.5 | 162365.63 | signal transduction | receptor protein kinase-like | EAZ45334.1 |
| Zeastar-E10-B03 |  |  | 7.2 | 4912.5 | sterol metabolism | endosperm C-24 sterol methyltransferase | NP_001106071.1 |
| Zeastar-E10-C03 | 1.8 | 1.6 |  | 26750 | translation | ribosomal protein S3A | NP_001046578.1 |
| Zeastar-E10-C10 | 2.1 | 1.8 |  | 23165.63 | DNA folding | histone H2B | P30756 |
| Zeastar-E10-C12 |  | 1.8 | 60.4 | 26912.5 | translation | 40S ribosomal protein S19 | ABF96702.1 |
| Zeastar-E10-D12 |  |  | 3.1 | 9634.38 | transport | transmembrane protein FT27 | EAZ43426.1 |
| Zeastar-E10-E04 |  | 1.8 | 4.5 | 89159.38 | seed storage protein | zein | NP_001105888.1 |
| Zeastar-E10-F04 | 1.8 |  |  | 50962.5 | seed storage protein | 15 kDa β-zein | NP_001106004.1 |
| Zeastar-E10-G04 |  |  | 6.8 | 1103.13 | DNA folding | histone H3 | P68427 |
| Zeastar-E10-G07 |  | 1.9 | 31 | 70625 | energy metabolism | pyrophosphatase (**EC 3.6.1.1**) | NP_001054459.1 |
| Zeastar-E13-A06 | 1.9 |  |  | 7312.5 | AA metabolism | putative aminotransferase | EAZ31971.1 |
| Zeastar-E13-C09 |  |  | 0.5 | 12693.75 | DNA folding | histone H3 | CAL55839.1 |
| Zeastar-E13-F10 | 1.9 |  |  | 4890.63 | translation | 60S ribosomal protein L7A | ABR25690.1 |
| Zeastar-E14-A12 |  |  | 0.5 | 2859.38 | protein turnover | ubiquitin-like | EAZ44763.1 |
| Zeastar-E14-D11 | 0.3 |  |  | 3021.88 | AA metabolism | lysyl-tRNA synthetase (**EC 6.1.1.6**) | ABF97357.1 |
| Zeastar-E14-F02 |  |  | 4.1 | 2437.5 | transport | hexose transporter | AAF74568.1 |
| Zeastar-E14-G10 | 2.8 |  |  | 834.38 | protein folding | cyclophilin A-3 (**EC 5.2.1.8**) | P21569 |
| Zeastar-E14-H04 |  | 1.9 | 66 | 3834.38 | signal transduction | polyprotein ATP-binding serine protease | NP_569138.1 |
| Zeastar-E15-C10 |  |  | 2.5 | 14640.63 | carbohydrate metabolism | α-galactosidase (**EC 3.2.1.22**) | NP_001064939.1 |
| Zeastar-E15-D03 |  |  | 0.5 | 3268.75 | AA metabolism | alanine aminotransferase (**EC 2.6.1.2**) | AAC62456.1 |
| Zeastar-E15-D08 |  | 1.9 |  | 4153.13 | signal transduction | RAN (small GTP-binding protein) | NP_001056390.1 |
| Zeastar-E15-F06 | 1.9 |  |  | 2868.75 | translation | ribosomal protein L15 | EAY97341.1 |
| Zeastar-E15-F07 | 1.8 |  |  | 60793.75 | transport | chloroplast protein - translocon-like protein | NP_001048155.1 |
| Zeastar-E15-G06 |  | 2 | 90.6 | 14653.13 | translation | 60S ribosomal protein L36 | AAV83991.1 |
| Zeastar-E16-A02 |  | 1.6 |  | 10365.63 | DNA folding | histone H2B | P30755 |
| Zeastar-E16-A08 |  |  | 0.5 | 278.13 | AA metabolism | s-adenosylmethionine decarboxylase. (**EC 4.1.1.50**) | NP_001105713.1 |
| Zeastar-E16-C08 | 0.3 |  |  | 5893.75 | carbohydrate metabolism | similarity to endo-1 | NP_001055474.1 |
| Zeastar-E16-C09 | 2.1 | 1.6 | 7.6 | 4109.38 | AA metabolism | anthranilate synthase β subunit (**EC 4.1.3.27**) | EAZ30999.1 |
| Zeastar-E16-D01 | 2.1 |  |  | 14815.63 | cytoskeleton | putative ARP protein | NP_001051041.1 |
| Zeastar-E16-D08 | 1.8 |  |  | 4937.5 | transcription | putative transcription initiation factor IIe | EAY78372.1 |
| Zeastar-E16-D10 | 1.9 |  |  | 1900 | translation | ribosomal protein L11 | NP_001046386.1 |
| Zeastar-E16-E08 | 2.2 |  |  | 3384.38 | signal transduction | serine/threonine-protein kinase | NP_001105274.1 |
| Zeastar-E16-F01 |  | 1.6 | 2.7 | 2118.75 | carbohydrate metabolism | γ-aminobutyrate transaminase subunit precursor (**EC 2.6.1.19**) | NP_001053863.1 |
| Zeastar-E16-G12 |  | 2 | 11.7 | 5693.75 | energy metabolism | putative vacuolar atp synthase subunit D (**EC 3.6.3.14**) | EAY74727.1 |
| Zeastar-E16-H11 |  | 0.4 |  | 2593.75 | signal transduction | D-type cyclin | EAZ04741.1 |
| Zeastar-E17-B03 |  | 1.8 | 11.8 | 115825 | translation | 60S ribosomal protein L31 | NP_001047935.1 |
| Zeastar-E17-C09 | 2.2 |  |  | 43690.63 | energy metabolism | thioredoxin (**EC 1.8.1.9**) | NP_001105811.1 |
| Zeastar-E17-F12 | 3.3 | 1.8 |  | 6418.75 | carbohydrate metabolism | succinate dehydrogenase subunit (**EC 1.3.5.1**) | EAZ38613.1 |
| Zeastar-E18-A04 |  | 1.7 | 7 | 52296.88 | signal transduction | cell cycle switch protein | EAY88331.1 |
| Zeastar-E18-D07 |  | 1.8 | 8.5 | 13065.63 | sulfate metabolism | ATP sulfurylase | NP_001104877.1 |
| Zeastar-E18-E02 | 1.8 |  |  | 19909.38 | cytoskeleton | actin depolymerizing factor | NP_001105474.1 |
| Zeastar-E18-E08 | 1.9 | 1.9 | 63.1 | 20034.38 | energy metabolism | inducible phenylalanine ammonia-lyase (**EC 4.3.1.5**) | EAY86763.1 |
| Zeastar-E18-F09 | 1.9 |  |  | 127521.88 | cell wall | putative pectinacetyl esterase | NP_001060382.1 |
| Zeastar-E18-H11 |  | 0.7 | 0.4 | 829.38 | AA metabolism | putative asparaginyl-trna synthetase. (**EC 6.1.1.22**) | EAZ11937.1 |
| Zeastar-E19-C12 | 1.8 |  |  | 9453.13 | transcription factor | MADS box protein | NP_001105155.1 |
| Zeastar-E19-D03 |  | 1.9 | 73.1 | 3271.88 | signal transduction | serine/threonine-protein kinase | EAY72920.1 |
| Zeastar-E19-E03 | 1.9 |  |  | 2643.75 | transport | putative amino acid transport protein | EAY87023.1 |
| Zeastar-E19-E09 | 1.9 | 1.9 | 20.2 | 2437.5 | AA metabolism | cytosolic glutamine synthetase (**EC 6.3.1.2**) | P38559 |
| Zeastar-E19-E11 |  | 0.7 | 0.3 | 7953.13 | energy metabolism | thiosulfate sulfurtransferase (**EC 2.8.1.1**) | EAY83836.1 |
| Zeastar-E19-F04 |  |  | 3.4 | 456.25 | translation | 60S ribosomal protein L7A | NP_001061550.1 |
| Zeastar-E19-G07 | 2 |  |  | 1609.38 | translation | putative ribosomal protein S10 | NP_001045480.1 |
| Zeastar-E19-H05 |  | 2 | 63.6 | 6118.75 | signal transduction | putative ser/thr protein phosphatase | NP_001043937.1 |
| Zeastar-E20-A10 | 2.2 |  |  | 37762.5 | molecular chaperonine | DNAJ-related protein | AAC08009.1 |
| Zeastar-E20-A11 |  |  | 0.5 | 8790.63 | protein turnover | putative serine peptidase | NP_001065039.1 |
| Zeastar-E20-A12 | 1.7 |  |  | 5834.38 | AA metabolism | 3-hydroxyisobutyrate dehydrogenase (**EC 1.1.1.31**) | NP_001044212.1 |
| Zeastar-E20-C03 | 1.8 | 1.9 | 72.4 | 4603.13 | protein turnover | 26S proteasome regulatory particle | EAZ22822.1 |
| Zeastar-E20-D08 |  | 1.7 | 7.1 | 7478.13 | transcription | histone deacetylase 2 | Q9M4U4 |
| Zeastar-E20-F04 |  |  | 2.9 | 25937.5 | transcription factor | transcription factor vsf-1 | EAZ07858.1 |
| Zeastar-E20-F06 | 1.8 | 1.6 |  | 7600 | signal transduction | RAS-related GTP-binding protein | NP_001055087.1 |
| Zeastar-E20-G04 | 1.9 |  |  | 18812.5 | fatty-acid metabolism | acetyl-coA c-acyltransferase (**EC 2.3.1.16**) | EAY78786.1 |
| Zeastar-E21-A03 | 2.1 |  |  | 5068.75 | protein turnover | putative β6 proteasome subunit | NP_001063603.1 |
| Zeastar-E21-A04 | 1.7 | 1.8 | 4.5 | 4565.63 | seed storage protein | 27 kDa storage protein | NP_001105354.1 |
| Zeastar-E21-A12 |  | 2 | 10.8 | 2531.25 | seed storage protein | 19 kDa α-zein | NP_001105888.1 |
| Zeastar-E21-C09 | 1.9 | 1.8 | 31.8 | 384.38 | seed storage protein | 22 kDa α-zein | P04699 |
| Zeastar-E22-B05 | 1.7 | 1.6 | 8.5 | 15600 | signal transduction | embryo-specific protein 1 | EAY89082.1 |
| Zeastar-E22-C09 |  |  | 2.8 | 138340.63 | electron transport | cytochrome P450 | Q43255 |
| Zeastar-E22-D08 |  | 2 | 19.3 | 5103.13 | protein turnover | putative ubiquitin protein | EAZ16853.1 |
| Zeastar-E22-E08 | 1.7 | 1.6 | 56.6 | 4678.13 | transport | putative amino acid transport protein | CAE03099.2 |
| Zeastar-F01-D08 | 2.2 |  |  | 5781.25 | DNA folding | putative histone H2A | NP_001105357.1 |
| Zeastar-F01-E09 | 3.3 |  |  | 7521.88 | energy metabolism | NADH dehydrogenase (**EC 1.6.5.3**) | EAZ04909.1 |
| Zeastar-F01-E10 |  |  | 0.4 | 52621.88 | biosynthesis of steroids | farnesyl pyrophosphate synthase (**EC 2.5.1.1**) | NP_001105039.1 |
| Zeastar-F01-E17 | 1.9 | 1.8 | 0.5 | 23812.5 | nucleotide metabolism | putative uracil phosphoribosyltransferase | EAZ18025.1 |
| Zeastar-F01-K12 |  | 1.7 | 4.5 | 3868.75 | signal transduction | auxin response factor | EAZ32372.1 |
| Zeastar-F01-L05 |  |  | 0.5 | 51368.75 | transcription | JAB1 protein | NP_001054112.1 |
| Zeastar-F02-C14 |  |  | 2.6 | 1184.38 | carbohydrate metabolism | enolase (**EC 4.2.1.11**) | NP_001105896.1 |
| Zeastar-F02-D09 |  |  | 8.1 | 52815.63 | carbohydrate metabolism | phosphoglucomutase (**EC 5.4.2.2**) | NP_001105405.1 |
| Zeastar-F02-E14 | 1.7 |  |  | 1681.25 | carbohydrate metabolism | cytoplasmic aldolase | NP_001105336.1 |
| Zeastar-F04-D20 | 2 |  |  | 1618.75 | stress - defense response | heat-shock protein | Q08277 |
| Zeastar-F04-E02 | 2.2 |  |  | 59296.88 | translation | putative ribosomal protein L26 | AAC64166.1 |
| Zeastar-F04-F24 |  | 0.6 |  | 2956.25 | stress - defense response | heat shock protein | NP_001104935.1 |
| Zeastar-F04-G14 | 1.7 |  |  | 9712.5 | protein turnover | putative β6 proteasome subunit | NP_001063603.1 |
| Zeastar-F04-J14 |  | 1.7 |  | 47996.88 | translation | translational initiation factor | AAY33860.1 |
| Zeastar-F04-K04 |  |  | 2.6 | 923.13 | transport | potassium transporter | AAK53760.1 |
| Zeastar-F04-L17 |  |  | 8.7 | 25700 | transcription factor | opaque-2 | NP_001105421.1 |
| Zeastar-F04-L20 | 1.7 |  | 3.3 | 37225 | protein turnover | protease I | NP_001054220.1 |
| Zeastar-F04-M05 |  | 1.6 |  | 9831.25 | stress - defense response | putative heat-shock protein | NP_001042210.1 |
| Zeastar-F04-M10 |  | 1.9 | 9.1 | 33265.63 | signal transduction | auxin-binding protein | NP_001105353.1 |
| Zeastar-F04-N14 | 2 |  |  | 2500 | signal transduction | similarity to glutathione-s-transferase | CAH67930.1 |
| Zeastar-F05-B21 |  | 1.9 |  | 16281.25 | carbohydrate metabolism | D-TDP-glucose dehydratase (**EC 4.2.1.46**) | CAC14890.1 |
| Zeastar-F05-D15 |  | 1.7 | 2.1 | 6496.88 | seed storage protein | 27 kDa storage protein | AAA33537.1 |
| Zeastar-F05-F17 |  |  | 2.9 | 23103.13 | electron transport | putative cytochrome P450 | NP_001043634.1 |
| Zeastar-F05-F23 | 2 |  |  | 5306.25 | folding - stress response | 101 kDa heat shock protein | EAZ34966.1 |
| Zeastar-F05-H24 | 1.7 | 1.7 |  | 91962.5 | fatty-acid metabolism | AIM1 protein | NP_001046536.1 |
| Zeastar-F05-I05 |  | 1.7 |  | 5353.13 | energy metabolism | putative plasma membrane proton ATPase (**EC 3.6.3.6**) | NP_001049178.1 |
| Zeastar-F05-J15 | 1.8 |  |  | 5287.5 | cytoskeleton | α-tubulin | NP_001049400.1 |
| Zeastar-F05-J16 | 1.8 |  |  | 50087.5 | translation | 60S ribosomal protein L34 | EAZ09088.1 |
| Zeastar-F05-M09 |  | 1.9 | 9.6 | 14046.88 | seed storage protein | zein | NP_001105888.1 |
| Zeastar-F05-M21 |  |  | 3.6 | 3337.5 | fatty-acid metabolism | acyl carrier protein | NP_001059204.1 |
| Zeastar-F05-P07 |  |  | 2.5 | 8306.25 | seed storage protein | 27 kDa storage protein | AAA33537.1 |
| Zeastar-F05-P11 |  |  | 2.1 | 5218.75 | nucleotide metabolism | uracil phosphoribosyltransferase-like protein | EAZ34543.1 |
| Zeastar-G01-B01 |  |  | 0.2 | 72309.38 | splicing | TIA-1 related protein | EAZ19129.1 |
| Zeastar-G01-B21 | 1.7 |  | 55.3 | 3828.13 | seed storage protein | 22 kDa α-zein | NP_001105999.1 |
| Zeastar-G01-C02 | 1.9 |  |  | 2215.63 | carbohydrate metabolism | starch synthase (**EC 2.4.1.21**) | AAD13341.1 |
| Zeastar-G01-L15 |  | 1.7 | 25 | 1493.75 | seed storage protein | 19 kDa α-zein | NP_001105888.1 |
| Zeastar-G01-L20 |  |  | 0.3 | 3321.88 | AA metabolism | putative aminopeptidaseaminopeptidase | BAD09617.1 |
| Zeastar-G01-M18 |  | 1.7 | 8 | 265.63 | seed storage protein | 19 kDa α-zein | ABV71935.1 |
| Zeastar-G01-P08 | 1.9 |  |  | 8384.38 | cytoskeleton | actin | NP_001051822.1 |
| Zeastar-G01-P22 |  |  | 3.8 | 17293.75 | AA metabolism | putative 3-isopropylmalate dehydrogenase (**EC 1.1.1.85**) | AAP50991.1 |
| Zeastar-G03-C13 |  | 2 | 9.7 | 2693.75 | seed storage protein | zein | NP_001105888.1 |
| Zeastar-G03-C20 |  | 1.6 | 6.5 | 23234.38 | seed storage protein | zein | P04703 |
| Zeastar-G03-G08 | 2.3 | 1.6 |  | 13912.5 | translation | 40S ribosomal protein S2 | CAO23572.1 |
| Zeastar-G03-J18 | 1.8 |  |  | 12453.13 | translation | putative 40S ribosomal protein | NP_001050539.1 |
| Zeastar-G03-L04 |  |  | 2.4 | 4437.5 | carbohydrate metabolism | pyruvate dehydrogenase E1 β−subunit (**EC 1.2.4.1**) | NP_001105506.1 |
| Zeastar-G03-P11 |  | 1.7 |  | 5290.63 | cell wall | glycine-rich RNA binding protein | AAM16011.1 |
| Zeastar-G03-P15 |  | 2.5 |  | 73325 | seed storage protein | 27 kDa storage protein | AAA33537.1 |
| Zeastar-G03-P16 | 1.8 |  |  | 2443.75 | translation | ribosomal protein S6 | NP_001105544.1 |
| Zeastar-G04-B12 |  | 1.6 |  | 12587.5 | seed storage protein | 27 kDa storage protein | NP_001105354.1 |
| Zeastar-G04-D05 |  | 1.6 |  | 9600 | seed storage protein | 27 kDa storage protein | AAA33537.1 |
| Zeastar-G04-D09 |  | 1.8 |  | 2443.75 | seed storage protein | 27 kDa storage protein | AAA33537.1 |
| Zeastar-G04-E08 |  | 1.8 |  | 150559.38 | seed storage protein | 27 kDa storage protein | AAA33537.1 |
| Zeastar-G04-F05 |  | 1.7 |  | 3737.5 | seed storage protein | 27 kDa storage protein | AAA33537.1 |
| Zeastar-G04-H11 | 1.9 |  |  | 11993.75 | translation | ribosomal protein S29 | EAZ28737.1 |
| Zeastar-G04-H14 |  | 2.1 |  | 10950 | seed storage protein | 27 kDa storage protein | AAA33537.1 |
| Zeastar-G04-N01 |  | 1.8 |  | 76187.5 | carbohydrate metabolism | glyoxalase I (**EC 4.4.1.5**) | NP_001055113.1 |
| Zeastar-G04-N20 |  | 1.9 | 6.8 | 48665.63 | cell wall | hydroxyproline-rich glycoprotein | EAY85293.1 |
| Zeastar-G05-B14 |  | 1.7 |  | 5653.13 | carbohydrate metabolism | putative pyruvate kinase (**EC 2.7.1.40**) | EAZ12974.1 |
| Zeastar-G05-C01 | 2.8 |  |  | 6284.38 | signal transduction | G protein-like protein | NP_001043910.1 |
| Zeastar-G05-E01 | 2.3 |  |  | 1018.75 | translation | 40S ribosomal protein S19 | ABR25439.1 |
| Zeastar-G05-E07 |  |  | 0.4 | 2884.38 | cytoskeleton | putative kinetochore protein | AAT09201.1 |
| Zeastar-G05-G02 |  | 1.6 |  | 16450 | cell wall | glycine-rich RNA-binding protein | P10979 |
| Zeastar-G05-I02 | 2.2 |  |  | 2762.5 | DNA folding | putative histone H2A | NP_001105357.1 |
| Zeastar-G05-K20 | 0.4 | 1.7 |  | 21615.63 | seed storage protein | zein | NP_001105884.1 |
| Zeastar-G05-L07 | 1.9 |  | 2 | 9846.88 | AA metabolism | cytosine-specific methyltransferase (**EC 2.1.1.37**) | AAC16389.1 |
| Zeastar-G05-M12 | 2.1 |  |  | 3478.13 | cell wall | glycine-rich RNA binding protein | AAM16011.1 |
| Zeastar-G05-M17 |  | 1.8 |  | 8246.88 | transcription factor | opaque-2 | AAA33489.1 |
| Zeastar-G05-N18 | 1.7 |  |  | 6025 | protein turnover | ubiquitin fusion protein | NP_001054720.1 |
| Zeastar-H01-A08 | 3.6 |  |  | 17790.63 | DNA folding | histone H2B-3 | A2WKP3 |
| Zeastar-H01-D07 | 2.5 | 1.9 | 65.7 | 3165.63 | seed storage protein | 22 kDa α-zein | ABV71962.1 |
| Zeastar-H01-F10 | 0.4 |  |  | 23140.63 | carbohydrate metabolism | putative hydroxymethylglutaryl-coA lyase (**EC 4.1.3.4**) | ABA95738.2 |
| Zeastar-H02-D12 | 1.9 | 2.1 | 70.2 | 7859.38 | seed storage protein | 22 kDa α-zein | NP_001105747.1 |
| Zeastar-H02-E12 |  | 1.6 | 7.7 | 32325 | seed storage protein | 50 kDa γ-zein | NP_001105053.1 |
| Zeastar-H02-F03 | 2 | 1.8 | 32.6 | 2565.63 | carbohydrate metabolism | pyruvate dehydrogenase E1 β-subunit (**EC 1.2.4.1**) | EAZ21234.1 |
| Zeastar-H03-D01 |  | 1.7 | 2 | 11759.38 | translation | putative ribosomal protein L19 | ABR25436.1 |
| Zeastar-H03-H04 |  |  | 8.7 | 8287.5 | transport | partial coat protein | CAJ33883.1 |
| Zeastar-H04-B01 |  | 1.9 |  | 3700 | seed storage protein | zein | AAA33539.1 |
| Zeastar-H04-B08 |  | 1.9 | 10.6 | 50521.88 | seed storage protein | zein | AAA33538.1 |
| Zeastar-H04-E01 | 1.7 |  |  | 8050 | seed storage protein | zein | P04702 |
| Zeastar-H04-E09 |  | 1.6 | 8.4 | 1334.38 | seed storage protein | 11 kDa methionine-rich protein | NP_001105936.1 |
| Zeastar-H04-H02 |  | 1.8 | 9 | 39487.5 | seed storage protein | zein | CAA24720.1 |
| Zeastar-H05-B03 |  | 0.5 | 0.4 | 887.5 | seed storage protein | 11 kDa methionine-rich protein | NP_001105936.1 |
| Zeastar-H05-C05 |  | 0.7 | 0.5 | 14593.75 | electron transport | cytochrome C | P00056 |
| Zeastar-H05-G06 |  | 1.7 |  | 17028.13 | cell wall | endo-1.4-β-glucanase cel1 | BAA94257.1 |
| Zeastar-H06-A03 | 0.3 | 0.6 | 3.8 | 2140.63 | seed storage protein | zein | NP_001105884.1 |
| Zeastar-H06-B05 | 0.4 |  |  | 3246.88 | nucleotide metabolism | putative dihydropyrimidinase | EAY76205.1 |
| Zeastar-H06-F11 | 0.4 |  |  | 1787.5 | seed storage protein | 50 kDa γ-zein | NP_001105053.1 |
| Zeastar-H06-G05 |  | 2 | 4.6 | 7343.75 | Signal Transduction | putative RAN binding protein | EAZ40394.1 |
| Zeastar-H07-B07 | 2.2 |  |  | 45881.25 | translation | elongation factor 1α | AAL79774.1 |
| Zeastar-H07-D05 |  | 1.8 | 3.4 | 140856.25 | seed storage protein | 19 kDa α-zein | ABV71980.1 |
| Zeastar-H07-F02 | 0.2 |  |  | 7903.13 | stress - defense response | putative pi starvation-induced protein | ABR25786.1 |
| Zeastar-H09-D12 |  | 1.8 | 10.4 | 2900 | protein turnover | ubiquitin / ribosomal protein S27A | AAA33519.1 |
| Zeastar-H10-A07 |  | 1.7 |  | 231193.75 | seed storage protein | zein | NP_001105884.1 |
| Zeastar-H10-B06 | 1.8 |  |  | 2837.5 | carbohydrate metabolism | pyruvate dehydrogenase E1 α-subunit (**EC 1.2.4.1**) | EAY92846.1 |
| Zeastar-H10-C09 | 0.4 | 0.6 | 2.8 | 15106.25 | cytoskeleton | putative katanin | NP_001043833.1 |
| Zeastar-H10-E02 |  | 1.9 | 2.5 | 7781.25 | translation | 60S ribosomal protein L15 | ABK93503.1 |
| Zeastar-H10-F10 | 0.4 | 0.7 | 4.5 | 3846.88 | AA metabolism | anthranilate phosphoribosyltransferase-like protein (**EC 2.4.2.18**) | EAZ03872.1 |
| Zeastar-H13-E08 |  | 1.7 | 4.7 | 155603.13 | seed storage protein | 50 kDa γ-zein | NP_001105053.1 |
| Zeastar-H13-H12 | 2 | 1.8 | 15.5 | 10556.25 | carbohydrate metabolism | starch branching enzyme IIb (**EC 2.4.1.18**) | ABO25741.1 |
| Zeastar-H14-A05 | 0.4 |  |  | 3643.75 | seed storage protein | 50 kDa γ-zein | NP_001105053.1 |
| Zeastar-H14-F07 |  | 2 | 2.1 | 19453.13 | signal transduction | auxin-binding protein | AAA33430.1 |
| Zeastar-H14-F09 |  |  | 16 | 48843.75 | translation | rRNA-glycosidase (**EC 3.2.2.22**) | AAW82712.1 |
| Zeastar-H18-A10 |  | 1.8 | 80.3 | 31562.5 | seed storage protein | 22 kDa α-zein | P04699 |
| Zeastar-H18-C11 |  | 2 | 56.5 | 54800 | seed storage protein | 19 kDa α-zein | ABV71938.1 |
| Zeastar-H18-E10 |  | 1.7 | 56.6 | 7509.38 | seed storage protein | 22 kDa α-zein | ABV71962.1 |
| Zeastar-H18-F10 |  | 2.1 | 36.7 | 7103.13 | structural | coat protein | ABL09403.1 |
| Zeastar-H18-H10 |  | 1.7 | 34.5 | 18059.38 | transport | transport protein subunit-like | EAY74575.1 |
| Zeastar-H19-A06 |  |  | 4.3 | 5262.5 | seed storage protein | 19 kDa α-zein | NP_001105056.1 |
| Zeastar-H19-A12 | 2.1 |  |  | 14534.38 | translation | translation initiation factor 5a | NP_001105606.1 |
| Zeastar-H19-D11 |  | 1.9 |  | 16037.5 | translation | ribosomal protein L14-like | BAD22765.1 |
| Zeastar-H19-E12 |  |  | 2.3 | 132934.38 | seed storage protein | 18 kDa δ-zein | NP_001105884.1 |
| Zeastar-H19-H12 |  |  | 8.4 | 11450 | seed storage protein | zein | AAR84080.1 |
| Zeastar-H21-F08 |  | 1.7 | 5.9 | 3300 | transcription | histone deacetylase 2 | NP_001105631.1 |
| Zeastar-H21-G08 |  |  | 11 | 7603.13 | seed storage protein | zein | AAR84080.1 |
| Zeastar-H23-A06 |  | 1.7 | 7.9 | 16712.5 | carbohydrate metabolism | alcohol dehydrogenase (**EC 1.1.1.1**) | AAB59302.1 |
| Zeastar-H23-B03 |  |  | 4.3 | 6990.63 | protein turnover | ubiquitin conjugating enzyme ligase | ABR25804.1 |
| Zeastar-H23-B12 |  |  | 6.1 | 1950 | protein turnover | putative ubiquitin protein | EAZ16853.1 |
| Zeastar-H23-D03 |  |  | 4.6 | 54525 | seed storage protein | zein | ABV71977.1 |
| Zeastar-H23-D08 |  |  | 9.9 | 2293.75 | seed storage protein | zein | AAA33539.1 |
| Zeastar-H23-D12 |  |  | 6.3 | 29371.88 | signal transduction | serine/threonine-protein kinase | EAZ00260.1 |
| Zeastar-H23-G03 |  | 1.9 | 75.4 | 8462.5 | seed storage protein | α-zein | AAK32953.1 |
| Zeastar-H23-G10 | 1.8 | 1.8 | 41.2 | 14425 | stress - defense response | putative cold-induced protein | NP_001056613.1 |
| Zeastar-H23-H06 |  |  | 3.8 | 19434.38 | translation | putative 40s ribosomal protein | ABR25600.1 |
| Zeastar-H24-A02 |  | 1.6 | 15.2 | 5337.5 | transport | mitochondrial energy transfer protein | NP_001105889.1 |
| Zeastar-H24-B10 |  |  | 8.8 | 226.25 | stress - defense response | heat shock protein | EAZ45147.1 |
| Zeastar-H24-B12 |  | 1.6 |  | 60925 | signal transduction | RAB5a protein | EAZ21313.1 |
| Zeastar-H24-C05 |  |  | 8.2 | 12137.5 | nucleotide metabolism | DAL1 protein | EAY95423.1 |
| Zeastar-H24-C10 |  |  | 8.9 | 12326.25 | signal transduction | serine/threonine-protein kinase | NP_001105342.1 |
| Zeastar-H24-D06 |  | 1.8 | 10.3 | 9143.75 | translation | putative translation initiation factor IF-2 | EAZ35481.1 |
| Zeastar-H24-D12 |  |  | 4.9 | 30650 | sterol metabolism | putative oxysterol-binding protein | EAZ26431.1 |
| Zeastar-H24-E03 |  | 1.7 | 6.1 | 14475 | energy metabolism | putative ubiquinol-cytochrome C reductase (**EC 1.10.2.2**) | NP_001065827.1 |
| Zeastar-H24-E07 |  | 1.6 | 5.3 | 11756.25 | translation | phenylalanine-trna synthetase-like | EAZ35247.1 |
| Zeastar-H24-F04 |  | 1.8 | 48.3 | 13043.75 | secondary metabolite metabolism | kaurene synthase | EAZ31953.1 |
| Zeastar-H24-G03 | 1.8 |  |  | 23959.38 | translation | ribosomal protein L38 | NP_001055990.1 |
| Zeastar-H24-G05 |  |  | 4.6 | 30003.13 | signal transduction | dual-specificity protein | NP_001043112.1 |
| Zeastar-H25-B03 |  |  | 7.4 | 10100 | translation | 60S acidic ribosomal protein P1-like | NP_001105701.1 |
| Zeastar-H25-B06 |  | 1.9 | 21.1 | 12237.5 | translation | 40S ribosomal protein S9 | EAZ25628.1 |
| Zeastar-H25-B09 |  | 1.7 | 4.5 | 13043.75 | carbohydrate metabolism | putative 2-isopropylmalate synthase (**EC 2.3.3.13**) | NP_001066116.1 |
| Zeastar-H25-D08 |  | 1.8 | 3.9 | 8959.38 | mRNA processing | novel cap-binding protein | NP_001049628.1 |
| Zeastar-H25-E02 |  | 1.7 | 6.6 | 46812.5 | energy metabolism | putative quinone oxidoreductase (**EC 1.6.5.3**) | CAO21187.1 |
| Zeastar-H25-F04 |  | 1.7 | 8.9 | 28234.38 | seed storage protein | 22 kDa α-zein | ABV71970.1 |
| Zeastar-H25-F12 |  | 1.7 | 5.5 | 10493.75 | seed storage protein | 19 kDa α-zein | ABV71939.1 |
| Zeastar-H25-G06 |  | 1.7 | 7.4 | 11031.25 | energy metabolism | putative aaa-metalloprotease (**EC 3.4.24.-**) | EAZ34562.1 |
| Zeastar-H25-H01 |  |  | 7.6 | 23556.25 | seed storage protein | zein | NP_001105746.1 |
| Zeastar-H26-A01 |  |  | 7.7 | 19287.5 | signal transduction | serine/threonine-protein kinase | CAA73067.1 |
| Zeastar-H26-A09 |  |  | 8.6 | 99934.38 | translation | threonyl-tRNA synthetaseaminoacyl-tRNA synthetase | EAZ23342.1 |
| Zeastar-H26-B08 |  | 1.6 | 4.9 | 10528.13 | translation | cytoplasmic ribosomal protein S13 | ABR25449.1 |
| Zeastar-H26-C06 |  |  | 2.5 | 2103.13 | transcription | histone acetyltransferase | EAZ31142.1 |
| Zeastar-H26-D12 | 0.3 |  |  | 7793.75 | energy metabolism | putative H^+^-transporting ATPase | CAO44156.1 |
| Zeastar-H26-F08 |  |  | 3 | 7606.25 | carbohydrate metabolism | putative glucosyltransferase (**EC 2.4.1.13**) | EAZ44804.1 |
| Zeastar-H26-G08 | 1.7 | 1.6 | 9.4 | 6031.25 | fatty-acid metabolism | acyl-coA synthetase (**EC 6.2.1.3**) | EAY75232.1 |
| Zeastar-H26-H03 |  |  | 4 | 93603.13 | signal transduction | putative auxin independent growth-related protein | EAZ10694.1 |
| Zeastar-H27-B09 |  |  | 11.8 | 19665.63 | fatty-acid metabolism | putative phytoene dehydrogenase (**EC 1.3.99.-**) | NP_001105381.1 |
| Zeastar-H27-B10 |  | 1.7 | 7.1 | 21359.38 | carbohydrate metabolism | putative fructose-bisphosphate aldolase (**EC 4.1.2.13**) | EAZ10324.1 |
| Zeastar-H27-C07 |  |  | 14.5 | 26190.63 | signal transduction | calcium-dependent serine/threonine-protein kinase | NP_001105306.1 |
| Zeastar-H27-D01 |  | 1.9 | 9 | 18106.25 | seed storage protein | zein | NP_001105888.1 |
| Zeastar-H27-D02 |  | 2 | 8.1 | 3321.88 | transport | putative ADP-ribosylation factor | NP_001065511.1 |
| Zeastar-H27-E07 |  |  | 4.6 | 1906.25 | translation | putative elongation factor 1b-γ | EAY85044.1 |
| Zeastar-H27-F12 |  | 1.8 | 15.9 | 15253.13 | translation | translation initiation factor | NP_001105606.1 |
| Zeastar-H27-G11 | 1.9 | 1.7 | 37.8 | 17825 | transport | partial coat protein | CAM32744.1 |
| Zeastar-H28-D02 |  | 1.8 | 35.2 | 2434.38 | stress - defense response | autophagy | AAY67885.1 |
| Zeastar-H28-F02 |  |  | 6.6 | 75937.5 | translation | ribosomal protein L15 | BAD22764.1 |
| Zeastar-H28-F05 | 0.6 |  |  | 5659.38 | seed storage protein | 10 kDa zein | NP_001105936.1 |
| Zeastar-H28-F07 |  | 2 | 68.1 | 7493.75 | seed storage protein | 22 kDa α-zein | AAK32952.1 |
| Zeastar-H28-G02 |  | 1.6 | 6 | 3640.63 | seed storage protein | 19 kDa α-zein | P02859 |
| Zeastar-H28-G08 |  | 1.6 | 8.9 | 13540.63 | seed storage protein | zein | P04703 |
| Zeastar-H28-H12 | 1.8 |  |  | 2262.5 | carbohydrate metabolism | putative phosphoglycerate mutase (**EC 5.4.2.1**) | NP_001105584.1 |
| Zeastar-H29-A03 |  | 1.9 |  | 140743.75 | AA metabolism | putative ketol-acid reductoisomerase (**EC 1.1.1.86**) | ABR25710.1 |
| Zeastar-H30-C04 | 1.8 | 1.8 | 50.7 | 4178.13 | carbohydrate metabolism | enolase (**EC 4.2.1.11**) | NP_001105371.1 |
| Zeastar-H30-C05 |  | 1.6 | 8.3 | 1968.75 | seed storage protein | zein | AAR84080.1 |
| Zeastar-H30-C11 |  | 1.7 | 74.7 | 346775 | seed storage protein | 22 kDa α-zein | AAC01577.1 |
| Zeastar-H30-E10 |  |  | 4.2 | 659396.88 | RNA modification | putative splicing factor | EAY88585.1 |
| Zeastar-H30-F06 |  | 1.8 | 10.1 | 4293.75 | seed storage protein | zein | CAA24720.1 |
| Zeastar-H30-F09 |  |  | 4.8 | 223.13 | transcription factor | hvb12d homologue | NP_001059360.1 |
| Zeastar-H30-G04 |  |  | 2.1 | 168075 | AA metabolism | putative alanine glyoxylate aminotransferase (**EC 2.6.1.2**) | EAY88729.1 |
| Zeastar-H30-H09 |  | 1.9 |  | 11396.88 | stress - defense response | thionin like protein | BAA95697.1 |
| Zeastar-H31-B04 |  |  | 3.1 | 7246.88 | protein folding | putative thioredoxin (**EC 5.3.4.1**) | EAZ23950.1 |
| Zeastar-H31-B11 |  |  | 10.2 | 38356.25 | AA metabolism | putative amino acid acetyltransferase (**EC 2.3.1.1**; **2.3.1.35**) | NP_001060125.1 |
| Zeastar-H31-C01 |  | 1.8 | 10.2 | 23028.13 | viral | viral polyprotein | ABG37681.1 |
| Zeastar-H31-C10 |  |  | 6.1 | 10325 | signal transduction | casein kinase II α-subunit | NP_001105632.1 |
| Zeastar-H31-C11 |  |  | 8.1 | 4650 | translation | 40S ribosomal protein S16 | NP_001065632.1 |
| Zeastar-H31-D05 |  | 1.6 | 8.9 | 107131.25 | translation | ribosomal protein L35A | AAL59231.1 |
| Zeastar-H31-D06 |  | 1.7 | 9.9 | 2131.25 | translation | ribosomal protein L35A | AAL59231.1 |
| Zeastar-H31-E06 | 0.6 | 1.7 | 5.5 | 48628.13 | seed storage protein | zein | NP_001105888.1 |
| Zeastar-H32-A03 | 1.8 |  |  | 4553.13 | translation | ribosomal protein L35A | AAL59231.1 |
| Zeastar-H32-B06 | 2.5 |  |  | 2125 | translation | 40S ribosomal protein S15 | EDQ73967.1 |
| Zeastar-H32-C12 |  |  | 8.5 | 7725 | seed storage protein | zein | P04703 |
| Zeastar-H32-D07 |  |  | 6.7 | 3178.13 | seed storage protein | 19 kDa α-zein | AAL16985.1 |
| Zeastar-H32-E02 |  |  | 5.9 | 3490.63 | translation | putative 60S ribosomal protein L18A | NP_001056335.1 |
| Zeastar-H32-E09 |  | 1.8 | 6.2 | 13796.88 | seed storage protein | 19 kDa α-zein | P02859 |
| Zeastar-H32-E10 |  | 1.8 |  | 5065.63 | stress - defense response | putative universal stress protein USP1 | NP_001066983.1 |
| Zeastar-H32-E11 |  |  | 12.4 | 4418.75 | signal transduction | putative ser/thr protein kinase | EAZ41835.1 |
| Zeastar-H32-F11 |  | 1.7 | 8.6 | 7740.63 | seed storage protein | 10 kDa zein | AAA33541.1 |
| Zeastar-H32-H08 |  | 1.7 | 43.4 | 4915.63 | seed storage protein | 22 kDa zein | NP_001105057.1 |
| Zeastar-H33-C02 | 2.1 |  |  | 3093.75 | translation | 60S ribosomal protein L17 | EDQ79984.1 |
| Zeastar-H33-C04 |  |  | 6.2 | 15578.13 | carbohydrate metabolism | 2-oxoglutarate dehydrogenase (**EC 1.2.4.2**) | CAH66433.1 |
| Zeastar-H33-C08 | 0.4 | 0.6 | 3.6 | 29856.25 | seed storage protein | 18 kDa delta zein | NP_001105884.1 |
| Zeastar-H33-C10 |  |  | 10.9 | 5025 | signal transduction | ADP-ribosylation factor 1 | EAY92309.1 |
| Zeastar-H33-E09 |  |  | 12.1 | 5753.13 | fatty-acid metabolism | isopentenyl pyrophosphate (**EC 5.3.3.2**) | NP_001105037.1 |
| Zeastar-H34-A12 | 2.2 |  | 9.3 | 12718.75 | seed storage protein | 22 kDa α-zein | NP_001105939.1 |
| Zeastar-H34-B09 | 1.8 | 1.7 | 47.3 | 11740.63 | energy metabolism | carbonic anhydrase (**EC 4.2.1.1**) | AAA86944.1 |
| Zeastar-H34-C07 | 1.7 |  | 59.3 | 22165.63 | seed storage protein | zein | P04703 |
| Zeastar-H34-C10 | 1.8 |  | 7 | 2340.63 | seed storage protein | 19 kDa α-zein | AAL16985.1 |
| Zeastar-H34-C11 |  |  | 5.2 | 1235153.13 | molecular chaperonin | DNAj protein-like | ABK25685.1 |
| Zeastar-H34-E04 |  | 1.7 | 9.5 | 5284.38 | signal transduction | calcium-dependent serine/threonine-protein kinase | NP_001105542.1 |
| Zeastar-H34-F04 |  | 1.8 | 9.3 | 6603.13 | translation | L3 ribosomal protein | ABK63945.1 |
| Zeastar-H34-H10 | 1.9 | 1.6 | 54.2 | 15168.75 | transcription factor | HVB12D homolog | EAZ00341.1 |
| Zeastar-H35-A06 |  |  | 13.9 | 3928.13 | seed storage protein | zein | P04703 |
| Zeastar-H35-C07 |  | 1.9 | 12.4 | 5834.38 | seed storage protein | 22 kDa zein | NP_001105057.1 |
| Zeastar-H35-C10 |  | 1.7 | 10.7 | 4706.25 | signal transduction | serine/threonine-protein kinase | BAD19066.1 |
| Zeastar-H35-E04 |  |  | 10 | 1846.88 | structural | coat protein | ABG37673.1 |
| Zeastar-H35-E07 |  |  | 57.6 | 3396.88 | seed storage protein | 22 kDa α-zein | ABV71962.1 |
| Zeastar-H35-E08 |  |  | 11.7 | 5231.25 | xenobiotic biodegradation | epoxide hydrolase (**EC 3.3.2.3**) | NP_001056192.1 |
| Zeastar-H35-F02 |  |  | 6.7 | 3184.38 | carbohydrate metabolism | starch branching enzyme I precursor | AAO20100.1 |
| Zeastar-H35-F05 |  | 1.8 | 14.2 | 4140.63 | translation | putative 60S ribosomal protein L9 | NP_001045607.1 |
| Zeastar-H35-G01 |  |  | 13 | 12603.13 | signal transduction | glutathione s-transferase | AAA72758.1 |
| Zeastar-H35-H11 |  |  | 8.7 | 1853.13 | transcription - translation | ATP-dependent RNA helicase-like | EAY89752.1 |
| Zeastar-H36-E03 |  |  | 4 | 4034.38 | stress - defense response | autophagy 12A | Q1SF86 |
| Zeastar-H36-E04 |  |  | 2.2 | 2684.38 | stress - defense response | BETL4 protein | NP_001105122.1 |
| Zeastar-H36-G03 | 0.4 | 0.7 | 3.8 | 16759.38 | seed storage protein | 18 kDa δ-zein | AAC49069.1 |
| Zeastar-H36-G10 |  |  | 2 | 8906.25 | protein folding | peroxiredoxin (**EC 1.11.1.15**) | NP_001043845.1 |
| Zeastar-H36-G12 |  |  | 9.9 | 91818.75 | seed storage protein | zein | P04703 |
| Zeastar-H36-H12 |  |  | 2.7 | 549259.38 | AA metabolism | cysteine synthase (**EC 2.5.1.47**) | EAY99706.1 |
| Zeastar-H39-A12 | 1.8 |  | 3.2 | 406953.13 | signal transduction | putative GTP-binding protein | EAY91631.1 |
| Zeastar-H39-B07 | 3.4 |  |  | 1475 | carbohydrate metabolism | dihydrolipoamide s-acetyltransferase (**EC 2.3.1.12**) | NP_001057705.1 |
| Zeastar-H39-C07 | 1.8 |  |  | 2915.63 | translation | 60S ribosomal protein L7 | NP_001053797.1 |
| Zeastar-H39-C10 | 2.1 |  |  | 801553.13 | AA metabolism | tryptophan synthase (**EC 4.2.1.20**) | EAZ38915.1 |
| Zeastar-H39-D08 |  |  | 0.5 | 9037.5 | transport | SEC13p | NP_001045820.1 |
| Zeastar-H39-F10 | 2.7 |  |  | 5187.5 | translation | ribosomal protein L22 | NP_001050078.1 |
| Zeastar-H39-G02 | 2.3 | 1.6 |  | 19409.38 | translation | 40S ribosomal protein S23 | EDQ51524.1 |
| Zeastar-H39-G11 | 1.7 |  | 2.8 | 8196.88 | translation | putative 40S ribosomal protein | EAZ14059.1 |
| Zeastar-H40-B07 | 2.3 |  |  | 4534.38 | translation | putative 40S ribosomal protein | EAZ14059.1 |
| Zeastar-H41-C06 |  | 1.9 |  | 9862.5 | signal transduction | ADP-ribosylation factor | EDQ70400.1 |
| Zeastar-H41-E08 | 1.9 |  |  | 4331.25 | stress - defense response | putative cold-induced protein | NP_001056613.1 |
| Zeastar-H41-F01 | 1.7 |  | 2.1 | 8109.38 | viral | viral polyprotein | ABI75205.1 |
| Zeastar-H41-F10 | 2.2 |  |  | 5859.38 | translation | elongation factor 2 | NP_001046972.1 |
| Zeastar-H41-G07 | 2.8 |  |  | 4859.38 | carbohydrate metabolism | glyoxalase I (**EC 4.4.1.5**) | NP_001105217.1 |
| Zeastar-H42-C03 | 1.9 | 2.4 | 3.2 | 43746.88 | carbohydrate metabolism | sucrose phosphate synthase (**EC 2.4.1.14**) | NP_001105694.1 |
| Zeastar-H42-C11 | 2.5 |  |  | 15062.5 | translation | putative cytoplasmic ribosomal protein S15A | ABA46758.1 |
| Zeastar-H43-E12 | 2.8 | 1.6 |  | 3731.25 | translation | elongation factor 2 | CAO23809.1 |
| Zeastar-H44-A10 |  | 1.6 | 6.1 | 243.75 | folding - stress response | 10 kDa chaperonin | AAB63591.1 |
| Zeastar-H44-D02 |  | 1.9 | 78.5 | 3871.88 | seed storage protein | 22 kDa α-zein | AAK32953.1 |
| Zeastar-H44-D09 |  |  | 55.4 | 5421.88 | translation | ribosomal protein L35-like | EAZ30442.1 |
| Zeastar-H45-H12 | 2 | 1.7 |  | 3737.5 | translation | 40S ribosomal protein S14 | AAO41731.1 |
| Zeastar-H46-B01 |  |  | 2.1 | 13037.5 | translation | 40S ribosomal protein S23 | EDQ51524.1 |
| Zeastar-H47-C10 | 1.7 |  |  | 16565.63 | carbohydrate metabolism | phosphoenolpyruvate carboxylase (**EC 4.1.1.31**) | CAC85932.1 |
| Zeastar-H47-D09 | 2 |  |  | 13196.88 | translation | 60S ribosomal protein L13E | NP_001056613.1 |
| Zeastar-H47-F09 | 2.1 |  |  | 96568.75 | signal transduction | IAA-amino acid conjugate hydrolase-like protein | EAZ03382.1 |
| Zeastar-H47-G12 | 2.5 | 1.9 |  | 2390.63 | transcription factor | HMGD1 protein | NP_001105649.1 |
| Zeastar-H49-A12 |  |  | 3.3 | 4562.5 | translation | 60S ribosomal protein L27 | NP_001046570.1 |
| Zeastar-H49-B11 |  |  | 2.3 | 5250 | signal transduction | protein phosphatase 2a | AAM94368.1 |
| Zeastar-H49-D04 |  | 1.9 | 0.4 | 43343.75 | molecular chaperonine | von hippel-lindau binding protein | NP_001067032.1 |
| Zeastar-H49-F05 | 1.9 | 1.7 |  | 9325 | signal transduction | ADP-ribosylation factor 1 | EAY92309.1 |
| Zeastar-H49-F09 |  | 1.7 | 27.7 | 5568.75 | carbohydrate metabolism | alcohol dehydrogenase (**EC 1.1.1.1**) | AAB59302.1 |
| Zeastar-H49-G07 | 0.5 |  |  | 6753.13 | fatty-acid metabolism | caleosin | EAY89082.1 |
| Zeastar-H50-D01 |  | 1.7 | 10.8 | 66653.13 | DNA folding | histone H4 homologue | EAZ44836.1 |
| Zeastar-H50-D03 |  |  | 17.8 | 52531.25 | translation | putative 40S ribosomal protein | EAZ14059.1 |
| Zeastar-H50-E02 |  | 1.8 | 9 | 17584.38 | protein turnover | putative ubiquitin protein | EAZ16853.1 |
| Zeastar-H50-H07 | 2 |  |  | 17143.75 | carbohydrate metabolism | pyruvate decarboxylase (**EC 4.1.1.1**) | A2XFI3.2 |
| Zeastar-H51-E05 |  |  | 58.5 | 19062.5 | carbohydrate metabolism | starch branching enzyme IIb (**EC 2.4.1.18**) | AAC33764.1 |
| Zeastar-H51-F05 | 1.8 |  |  | 1023.13 | protein turnover | ubiquitin-protein ligase 2 | EAZ43920.1 |
| Zeastar-H52-A03 |  |  | 9.4 | 59187.5 | signal transduction | response regulator phosphorylation | BAE79358.1 |
| Zeastar-H52-H05 | 1.7 |  |  | 10721.88 | protein turnover | ubiquitin conjugating protein | CAO62520.1 |
| Zeastar-H52-H06 |  |  | 5.7 | 4309.38 | transcription | histone deacetylase hda101 | NP_001105064.1 |
| Zeastar-I01-C03 | 2 |  |  | 15006.25 | translation | 60S ribosomal protein L24 | NP_001055880.1 |
| Zeastar-I01-D03 |  | 1.7 |  | 2915.63 | signal transduction | putative glycosylphosphatidylinositol-anchored protein | NP_001105969.1 |
| Zeastar-I04-H03 |  | 2 | 81.8 | 229734.38 | seed storage protein | 22 kDa α-zein | NP_001105747.1 |
| Zeastar-I04-H06 | 1.9 | 1.6 | 74 | 16368.75 | seed storage protein | 22 kDa α-zein | AAC01576.1 |
| Zeastar-I05-D09 |  |  | 0.2 | 5293.75 | RNA modification | s-like RNAse | NP_001106070.1 |
| Zeastar-I06-D05 | 1.7 | 1.7 | 4.2 | 59278.13 | carbohydrate metabolism | similarity to ENDO-1 | NP_001104915.1 |
| Zeastar-I06-D07 |  |  | 3.3 | 10340.63 | storage protein | patatin-like protein | AAD22170.1 |
| Zeastar-I06-G05 | 2 |  |  | 5787.5 | transcription factor | yabby2 | EAY83940.1 |
| Zeastar-I07-A10 |  |  | 40.2 | 10956.25 | seed storage protein | 22 kDa α-zein | NP_001105058.1 |
| Zeastar-I07-D10 |  | 2 | 4.5 | 2450 | seed storage protein | 19 kDa α-zein | NP_001105056.1 |
| Zeastar-L01-A08 |  |  | 5.4 | 1831.25 | signal transduction | cyclin H-1 | AAU93531.1 |
| Zeastar-L01-D01 |  | 1.8 | 16 | 5865.63 | seed storage protein | 22 kDa α-zein | ABV71974.1 |
| Zeastar-L01-E03 |  | 1.9 | 17.4 | 41284.38 | carbohydrate metabolism | pyruvate dehydrogenase (**EC 2.3.1.12**) | EAY90179.1 |
| Zeastar-L02-F02 |  |  | 6.7 | 6693.75 | seed storage protein | 19 kDa zein | CAA47640.1 |
| Zeastar-L03-D08 |  | 1.7 | 63.6 | 7468.75 | seed storage protein | 22 kDa zein | NP_001105747.1 |
| Zeastar-L04-B01 |  | 1.9 | 9.6 | 2412.5 | seed storage protein | 19 kDa α-zein | P06674 |
| Zeastar-L04-C10 |  | 1.7 |  | 3290.63 | seed storage protein | 19 kDa α-zein | ABV71935.1 |
| Zeastar-M01-H10 |  | 1.6 |  | 4984.38 | transport | peptide transporter | NP_001041994.1 |
| Zeastar-M02-H11 |  |  | 2.2 | 13662.5 | stress - defense response | heat-shock protein ATP-binding | EAZ02096.1 |
| Zeastar-M04-A05 | 1.9 |  |  | 4253.13 | translation | 60S ribosomal protein L18A | NP_001056335.1 |
| Zeastar-M05-E09 |  | 1.9 |  | 1590.63 | nucleotide metabolism | putative small nuclear ribonucleoprotein | NP_001050345.1 |
| Zeastar-M13-B01 |  | 0.6 | 5.3 | 2593.75 | RNA modification | putative RNA methyltransferasesmethyltransferase | NP_001050924.1 |
| Zeastar-M13-H03 |  | 1.7 |  | 357571.88 | ribosome biogenensis | pescadillo-like protein | EAY91517.1 |
| Zeastar-M14-F09 | 2.1 |  |  | 5221.88 | translation | 40S ribosomal protein S15 | EAZ38933.1 |
| Zeastar-M15-B12 |  | 1.6 |  | 58287.5 | AA metabolism | carbamoyl phosphate synthetase small subunit (**EC 6.3.5.5**) | EAZ24340.1 |
| Zeastar-M15-F01 |  |  | 4.6 | 7075 | transport | putative lipid transfer protein | NP_001105602.1 |
| Zeastar-M15-G03 |  | 1.9 | 56.6 | 41165.63 | protein turnover | ubiquitin-conjugating enzyme | CAI29540.1 |
| Zeastar-M15-H08 | 1.8 |  | 19.5 | 4156.25 | translation | 60S ribosomal protein L24 | NP_001055880.1 |
| Zeastar-M16-G11 |  | 1.8 |  | 2790.63 | energy metabolism | soluble inorganic pyrophosphatase (**EC 3.6.1.1**) | NP_001104889.1 |
| Zeastar-M19-E05 |  |  | 4.5 | 27990.63 | electron transport | electron transfer flavoprotein β-subunit | EAY93214.1 |
| Zeastar-N01-F10 | 2.6 |  |  | 23356.25 | AA metabolism | putative trap protein | EAZ02360.1 |
| Zeastar-N02-C04 | 3 |  |  | 2246.88 | AA metabolism | phosphoglycerate dehydrogenase (**EC 1.1.1.95**) | NP_001059330.1 |
| Zeastar-N02-E03 | 2.6 |  |  | 78678.13 | transport | nonspecific lipid-transfer protein | ABA33849.1 |
| Zeastar-N02-G11 |  | 3.8 |  | 3540.63 | AA metabolism | putative spermidine synthase. (**EC 2.5.1.16**) | NP_001105842.1 |
| Zeastar-N03-A05 | 1.9 | 2.2 |  | 263259.38 | cell wall | cellulose synthase-like protein | NP_001047983.1 |
| Zeastar-N03-B12 | 3.2 |  |  | 3421.88 | cofactor - vitamin metabolism | δ-aminolevulinic acid dehydratase (**EC 4.2.1.24**) | EAZ02284.1 |
| Zeastar-N03-C02 | 1.8 | 1.7 |  | 17068.75 | protein turnover | 26S proteasome regulatory particle triple-a ATPase subunit 1 | NP_001057037.1 |
| Zeastar-N03-D01 |  |  | 0.3 | 23790.63 | stress - defense response | γ-thionin | ABG78829.1 |
| Zeastar-N03-F03 | 2.5 | 1.7 |  | 6471.88 | membrane transport | ADP/ATP translocase | EAZ12893.1 |
| Zeastar-N03-F12 | 1.9 |  |  | 231300 | protein turnover | putative protease | NP_001046837.1 |
| Zeastar-N04-A02 |  | 1.7 |  | 28237.5 | signal transduction | calcium-dependent serine/threonine-protein kinase | NP_001062383.1 |
| Zeastar-N04-C04 | 2.9 |  |  | 6009.38 | energy metabolism | pyrophosphatase (**EC 3.6.1.1**) | EAZ21995.1 |
| Zeastar-N04-C06 | 1.7 | 1.7 |  | 1025790.63 | AA metabolism | putative amidase | NP_001068027.1 |
| Zeastar-N05-C08 |  | 1.6 |  | 16815.63 | signal transduction | tawin2 | BAB11740.1 |
| Zeastar-N05-F08 | 1.9 |  |  | 11028.13 | translation | elongation factor 2 | NP_001046972.1 |
| Zeastar-N05-H03 | 2.2 | 1.6 |  | 23353.13 | translation | translational initiation factor EIF-4a | NP_001104874.1 |
| Zeastar-N06-A08 | 2.3 |  |  | 6553.13 | transport | nonspecific lipid-transfer protein | ABA33847.1 |
| Zeastar-N06-B04 | 2.1 |  |  | 9828.13 | transcription - translation | putative ATP-dependent RNA helicase | EAZ24934.1 |
| Zeastar-N06-C07 | 1.8 |  |  | 2653.13 | transport | calcium-binding transporter-like protein | EAY99214.1 |
| Zeastar-N06-C08 |  | 1.8 |  | 1323.13 | protein turnover | putative ubiquitin fusion-degradation protein | EAZ31718.1 |
| Zeastar-N07-C01 | 2.3 |  |  | 14568.75 | translation | ribsomal protein S4 | AAB67831.1 |
| Zeastar-N07-E04 | 2 |  |  | 3593.75 | translation | protein synthesis initiation factor 4g | ABO15893.1 |
| Zeastar-N07-E06 |  | 1.6 |  | 89503.13 | cytoskeleton | actin-related protein ARP11 | NP_001047323.1 |
| Zeastar-N07-F04 | 1.9 |  |  | 4609.38 | AA metabolism | peroxidase precursor (**EC 1.11.1.7**) | NP_001045484.1 |
| Zeastar-N07-F08 | 2.3 |  |  | 4193.75 | stress - defense response | osr40c1 protein | ABF95726.1 |
| Zeastar-N07-G02 | 2.2 |  |  | 5643.75 | protein turnover | 26S proteasome regulatory particle triple-a ATPase subunit 2b | NP_001060719.1 |
| Zeastar-N07-G09 | 3.6 | 1.7 |  | 89428.13 | translation | ribsomal protein S4 | AAB67831.1 |
| Zeastar-N08-A07 | 1.7 |  |  | 3421.88 | energy metabolism | pyrophosphatase (**EC 3.6.1.1**) | CAG29370.1 |
| Zeastar-N08-A12 | 1.8 | 1.9 |  | 3687.5 | fatty-acid metabolism | lipoxygenase (**EC 1.13.11.12**) | AAD32243.1 |
| Zeastar-N08-D10 | 1.9 |  |  | 28493.75 | fatty-acid metabolism | acyl-[acyl-carrier protein] desaturase (**EC 1.14.99.6**) | EAZ30471.1 |
| Zeastar-N08-H05 | 2.6 | 2.2 |  | 280021.88 | translation | ribosomal protein L15 | NP_001050612.1 |
| Zeastar-N08-H06 | 1.7 |  | 3.8 | 6721.88 | translation | ribosomal protein L19 | AAI26716.1 |
| Zeastar-N08-H12 | 2.2 |  |  | 29031.25 | AA metabolism | aspartate-tRNA ligase-like protein | EAZ24217.1 |
| Zeastar-N09-A02 | 2.1 |  |  | 1568.75 | transcription factor | transcription factor GAMYB | A2WW87.1 |
| Zeastar-N09-D08 | 2.1 |  |  | 96300 | transport | putative vamp (vesicle-associated membrane protein) protein | EAY95107.1 |
| Zeastar-N10-B05 |  | 1.9 |  | 1309.38 | cell wall | glycine-rich RNA binding protein | AAM16011.1 |
| Zeastar-N10-B08 | 1.7 |  |  | 20081.25 | carbohydrate metabolism | glyoxalase I (**EC 4.4.1.5**) | NP_001105217.1 |
| Zeastar-N10-C03 |  | 1.7 |  | 11065.63 | transcription factor | OCL5 DNA-binding homeobox protein | NP_001053951.1 |
| Zeastar-N10-D07 | 1.9 |  |  | 10571.88 | carbohydrate metabolism | β galactosidase-like protein (**EC 3.2.1.23**) | NP_001045421.1 |
| Zeastar-N10-D09 | 3.3 |  | 3.3 | 31468.75 | AA metabolism | acetolactate synthase (**EC 2.2.1.6**) | Q41769 |
| Zeastar-N10-H05 |  | 1.6 |  | 406709.38 | translation | tyrosyl-tRNA synthetase | BAD10425.1 |
| Zeastar-N10-H08 |  |  | 4.5 | 41337.5 | energy metabolism | sulfite oxidase (**EC 1.8.3.1**) | ABI53846.1 |
| Zeastar-N11-A10 | 1.9 |  |  | 37631.25 | splicing | spl1-related protein | Q6Z8M8.1 |
| Zeastar-N11-A11 | 2 |  |  | 10815.63 | splicing | spl1-related2 protein | NP_001062227.1 |
| Zeastar-N11-C07 | 1.7 | 1.6 |  | 4562.5 | translation | cytoplasmic ribosomal protein S13 | NP_001105708.1 |
| Zeastar-N11-D12 | 2.1 |  |  | 3418.75 | DNA folding | SGT1 | AAL33610.1 |
| Zeastar-N11-E10 |  | 1.8 |  | 257353.13 | protein folding | mitochondrial processing peptidase α-chain | EAZ34992.1 |
| Zeastar-N11-F04 | 2.2 |  |  | 38000 | stress - defense response | heat shock transcription factor | NP_001063364.1 |
| Zeastar-N11-F09 |  | 0.6 | 0.3 | 14475 | carbohydrate metabolism | chitinase-b (**EC 3.2.1.14**) | AAT40050.1 |
| Zeastar-N12-A12 | 2.2 |  |  | 323.13 | carbohydrate metabolism | NADP-specific isocitrate dehydrogenase (**EC 1.1.1.42**) | NP_001043749.1 |
| Zeastar-N12-C10 | 2.8 |  |  | 6159.38 | cytoskeleton | α tubulin subunit | NP_001105440.1 |
| Zeastar-N12-C12 | 1.9 |  |  | 48540.63 | carbohydrate metabolism | NADP-dependent malic enzyme (**EC 1.1.1.39**) | AAK91502.1 |
| Zeastar-N12-D03 | 2.2 | 1.7 |  | 2434.38 | cofactor – vitamin metabolism | ferrochelatase (**EC 4.99.1.1**) | NP_001062803.1 |
| Zeastar-N12-D11 | 2.8 |  |  | 1390.63 | transcription | putative cell division protein | NP_001058321.1 |
| Zeastar-N12-D12 | 1.9 |  |  | 2440.63 | membrane transport | ADP/ATP translocase | EAZ12893.1 |
| Zeastar-N13-A03 | 2.1 | 2.2 |  | 10337.5 | splicing | splicing factor-like protein | EAZ16209.1 |
| Zeastar-N13-F09 | 1.9 |  |  | 2315.63 | translation | putative eukaryotic release factor | EAY95388.1 |
| Zeastar-N13-F10 | 2 |  |  | 4565.63 | translation | elongation factor 1β2 | EAZ04699.1 |
| Zeastar-N14-A12 |  | 1.7 |  | 1296.88 | transport | putative HAK2 | BAD87252.1 |
| Zeastar-N14-B07 |  | 1.7 |  | 8225 | signal transduction | protein phosphatase 2a hydrolase | BAD17175.1 |
| Zeastar-N14-B09 |  | 1.6 |  | 15996.88 | fatty-acid metabolism | abc transporter | BAD09728.1 |
| Zeastar-N14-E01 |  | 1.8 |  | 1681.25 | signal transduction | serine/threonine-protein kinase | NP_001047841.1 |
| Zeastar-N14-F02 | 2.2 | 1.7 | 27.5 | 26443.75 | cell wall | cellulose synthase-like protein | EAY87358.1 |
| Zeastar-N14-H11 | 2.1 |  |  | 4256.25 | carbohydrate metabolism | transaldolase (**EC 2.2.1.2**) | NP_001061014.1 |
| Zeastar-N15-A05 | 1.8 | 1.7 |  | 10868.75 | translation | putative eukaryotic translation initiation factor | NP_001105726.1 |
| Zeastar-N15-E07 | 2.3 | 1.9 |  | 12778.13 | translation | 40S ribosomal protein S9 | NP_001049004.1 |
| Zeastar-N15-G05 | 1.7 |  |  | 8581.25 | AA metabolism | putative phosphoethanolamine n-methyltransferase | EAZ13191.1 |
| Zeastar-N16-A01 |  | 1.9 |  | 3540.63 | transport | voltage-dependent anion channel protein 2 | NP_001104949.1 |
| Zeastar-N16-E03 | 2.7 |  |  | 1403.13 | AA metabolism | s-adenosyl-l-methionine synthetase (**EC 2.5.1.6**) | CAJ45555.1 |
| Zeastar-N16-E07 | 1.8 |  |  | 6437.5 | RNA modification | RNAse l inhibitor-like protein | AAL26702.1 |
| Zeastar-N16-F02 | 3.1 |  |  | 3009.38 | translation | ribosomal protein S3A | EAZ22617.1 |
| Zeastar-N16-F09 | 2 |  |  | 2475 | stress - defense response | dolichyl-di-phosphooligosaccharide-protein glycotransferase | EAZ03190.1 |
| Zeastar-N16-G05 |  |  | 2.3 | 3181.25 | membrane | membrane spanning protein | EAZ23666.1 |
| Zeastar-N17-A02 | 2.3 |  |  | 8734.38 | transcription factor | transcription factor | NP_001048319.1 |
| Zeastar-N17-A05 | 2 |  |  | 24021.88 | transport | protein transport protein | NP_001046076.1 |
| Zeastar-N17-C06 | 2.7 | 1.8 |  | 4453.13 | translation | ribosomal protein | EAZ41605.1 |
| Zeastar-N17-C11 |  |  | 2.2 | 14612.5 | nucleotide metabolism | phosphoribosylformylglycinamidine cyclo-ligase | EAZ29171.1 |
| Zeastar-N17-C12 | 1.8 |  |  | 7143.75 | membrane | membrane glycoprotein | ABF93903.1 |
| Zeastar-N17-D03 | 2.5 |  |  | 19125 | membrane | major surface glycoprotein-like | NP_001051165.1 |
| Zeastar-N17-E09 | 2.2 |  |  | 10943.75 | AA metabolism | methionine synthase (**EC 2.1.1.14**) | ABK96186.1 |
| Zeastar-N17-F04 | 2.1 |  |  | 8290.63 | protein turnover | putative 26S proteasome p55 protein | EAZ29325.1 |
| Zeastar-N17-H02 |  | 1.7 |  | 8812.5 | signal transduction | putative cell cycle serine/threonine-protein kinase | EAZ16994.1 |
| Zeastar-N18-A04 | 2 |  |  | 5734.38 | translation | 60S ribosomal protein L34 | EAZ09088.1 |
| Zeastar-N18-F04 | 1.8 |  |  | 141737.5 | protein turnover | aspartic proteinase | P42210 |
| Zeastar-N18-G04 |  | 9.5 |  | 23890.63 | carbohydrate metabolism | chitinase b (**EC 3.2.1.14**) | AAT40038.1 |
| Zeastar-N19-B03 |  | 1.9 |  | 10500 | transcription factor | putative MYB family transcription factor | NP_001050406.1 |
| Zeastar-N19-B07 | 1.9 |  |  | 55325 | transport | plasma membrane MIP protein | NP_001104934.1 |
| Zeastar-N19-C02 |  |  | 2.3 | 9162.5 | transport | high-affinity potassium transporter | NP_001045288.1 |
| Zeastar-N19-C12 |  |  | 0.2 | 45596.88 | seed storage protein | vicilin-like embryo storage protein | CAA41809.1 |
| Zeastar-N19-D08 | 2 |  |  | 4228.13 | transport | coatomer delta subunit | NP_001104961.1 |
| Zeastar-N19-F04 |  | 1.7 |  | 14237.5 | fatty-acid metabolism | not56-like protein | EAY72720.1 |
| Zeastar-N19-F07 | 2.3 |  |  | 6025 | signal transduction | protein kinase ATN1 | NP_001047304.1 |
| Zeastar-N19-G02 | 2.6 |  |  | 51534.38 | translation | 40S ribosomal protein S24 | NP_001046358.1 |
| Zeastar-N20-A07 |  | 1.6 |  | 4709.38 | energy metabolism | ferredoxin-sulfite reductase precursor (**EC 1.8.7.1**) | NP_001105302.1 |
| Zeastar-N20-B01 | 2 |  |  | 10696.88 | fatty-acid metabolism | isopentenyl diphosphate delta isomerase (**EC 5.3.3.2**) | NP_001105275.1 |
| Zeastar-N20-C11 | 1.7 | 1.9 |  | 3371.88 | cell wall | type IIIa membrane protein cp-wap11 | NP_001105598.1 |
| Zeastar-N20-E01 | 2 |  |  | 3603.13 | carbohydrate metabolism | trehalose synthase (**EC 2.4.1.15**) | EAY75823.1 |
| Zeastar-N20-E07 | 1.7 |  |  | 1781.25 | carbohydrate metabolism | citrate synthase (**EC 2.3.3.1**) | EAY81201.1 |
| Zeastar-N20-F01 |  | 1.8 |  | 9665.63 | DNA replication | proliferating cell nuclear antigen | NP_001105404.1 |
| Zeastar-N20-F04 | 1.8 |  |  | 1778.13 | transport | porin-like protein | AAP46186.1 |
| Zeastar-N20-F10 | 1.8 |  |  | 44090.63 | signal transduction | putative casein kinase | EAY92152.1 |
| ZT-P10-1-A07 | 3 |  |  | 6909.38 | translation | putative elongation factor | NP_001046972.1 |
| ZT-P10-1-C12 | 3.5 | 1.9 |  | 2646.88 | signal transduction | activated protein kinase c receptor homolog | ABR25943.1 |
| ZT-P10-1-E01 | 2.6 |  |  | 9096.88 | carbohydrate metabolism | UDP-glucose dehydrogenase (**EC 1.1.1.22**) | EAY91919.1 |
| ZT-P10-1-G12 | 1.8 |  |  | 5496.88 | translation | ribosomal protein L7AE-like | CAO65649.1 |
| ZT-P10-2-A11 |  |  | 2.6 | 6690.63 | transcription | initiation factor 3g | EAY87804.1 |
| ZT-P10-2-C12 | 2.1 |  |  | 2390.63 | protein turnover | 26S proteasome regulatory subunit | EAZ41178.1 |
| ZT-P10-3-C05 | 2.9 | 1.9 |  | 309706.25 | DNA folding | histone H4 | NP_001070058.1 |
| ZT-P10-3-C10 |  | 1.8 |  | 55284.38 | energy metabolism | ATPase (**EC 3.6.3.14**) | EAZ38336.1 |
| ZT-P10-3-G03 | 1.9 |  |  | 13715.63 | carbohydrate metabolism | transaldolase (**EC 2.2.1.2**) | EAZ14690.1 |
| ZT-P10-4-C06 | 0.5 |  |  | 2712.5 | seed storage protein | 50 kDa γ-zein | NP_001105053.1 |
| ZT-P10-4-E12 |  |  | 2.5 | 25959.38 | carbohydrate metabolism | xylose isomerase (**EC 5.3.1.5**) | NP_001060585.1 |
| ZT-P10-4-G04 | 1.9 |  |  | 7871.88 | transport | voltage-dependent anion channel protein 1a | NP_001105619.1 |
| ZT-P10-5-B03 |  | 0.5 |  | 619628.13 | stress - defense response | wound-induced protease inhibitor | AAS17857.1 |
| ZT-P10-5-D05 | 2 |  |  | 4959.38 | translation | putative ribosomal protein S8 | NP_001052526.1 |
| ZT-P10-5-G07 | 2.3 |  |  | 57515.63 | translation | elongation factor 1-α | NP_001105587.1 |
| ZT-P10-6-B04 | 2 |  |  | 4496.88 | signal transduction | cysteine proteinase precursor | NP_001105685.1 |
| ZT-P10-6-D11 | 1.8 |  |  | 18228.13 | translation | ribosomal protein L22 | NP_001060613.1 |
| ZT-P10-6-G08 | 1.9 |  |  | 15493.75 | translation | ribosomal protein L5A | A2WXX3.2 |
| ZT-P10-7-C10 | 1.9 |  |  | 121571.88 | stress - defense response | putative heat shock protein | ABB84343.1 |
| ZT-P10-7-D03 |  | 0.5 |  | 1268.75 | stress - defense response | wound-induced protease inhibitor | AAL01270.1 |
| ZT-P10-7-F08 | 2.3 |  |  | 6315.63 | translation | translation initiation factor 4a2 | AAD20980.1 |
| ZT-P21-1-D02 |  | 1.6 |  | 7200 | structural | coat protein | ABI75204.1 |
| ZT-P21-3-A07 |  | 1.8 | 10.5 | 167287.5 | seed storage protein | zein | ABV71958.1 |
| ZT-P21-3-H03 |  | 1.6 | 6.4 | 88165.63 | signal transduction | cysteine proteinasehydrolase | NP_001105685.1 |
| ZT-P21-4-E10 | 2 |  |  | 14053.13 | carbohydrate metabolism | xylose isomerase (**EC 5.3.1.5**) | NP_001060585.1 |
| ZT-P21-4-F06 | 0.6 |  |  | 9237.5 | signal transduction | small GTP binding protein RAB2 | ABD59354.1 |
| ZT-P21-6-B12 |  | 2.1 | 17.1 | 6406.25 | seed storage protein | α−zein precursor | P06679 |
| ZT-P21-6-G11 |  | 1.6 |  | 5465.63 | transport | putative β-coat protein | EAY73532.1 |
| ZT-P21-7-A03 |  | 1.8 |  | 7925 | transport | partial coat protein | CAJ33883.1 |
| ZT-P21-7-F08 | 2 |  | 0.4 | 11131.25 | AA metabolism | s-adenosylmethionine decarboxylase (**EC 4.1.1.50**) | ABO32298.1 |
| ZT-P21-7-G09 | 2.8 |  |  | 9040.63 | stress - defense response | putative cold-induced protein | NP_001056613.1 |
